# Incorporation of the influenza A virus NA segment into virions does not require cognate non-coding sequences

**DOI:** 10.1038/srep43462

**Published:** 2017-02-27

**Authors:** Bernadette Crescenzo-Chaigne, Cyril V. S. Barbezange, Stéphane Léandri, Camille Roquin, Camille Berthault, Sylvie van der Werf

**Affiliations:** 1 Institut Pasteur, Unité de Génétique Moléculaire des Virus à ARN, Paris, France; 2 Unité Mixte de Recherche 3569, Centre National de la Recherche Scientifique, Paris, France; 3 Université Paris-Diderot Sorbonne-Paris-Cité, Paris, France

## Abstract

For each influenza virus genome segment, the coding sequence is flanked by non-coding (NC) regions comprising shared, conserved sequences and specific, non-conserved sequences. The latter and adjacent parts of the coding sequence are involved in genome packaging, but the precise role of the non-conserved NC sequences is still unclear. The aim of this study is to better understand the role of the non-conserved non-coding sequences in the incorporation of the viral segments into virions. The NA-segment NC sequences were systematically replaced by those of the seven other segments. Recombinant viruses harbouring two segments with identical NC sequences were successfully rescued. Virus growth kinetics and serial passages were performed, and incorporation of the viral segments was tested by real-time RT-PCR. An initial virus growth deficiency correlated to a specific defect in NA segment incorporation. Upon serial passages, growth properties were restored. Sequencing revealed that the replacing 5′NC sequence length drove the type of mutations obtained. With sequences longer than the original, point mutations in the coding region with or without substitutions in the 3′NC region were detected. With shorter sequences, insertions were observed in the 5′NC region. Restoration of viral fitness was linked to restoration of the NA segment incorporation.

In humans, influenza A viruses are responsible for yearly epidemics of respiratory tract infections that represent a constant burden for national health services worldwide^1^. The introduction of a new influenza virus into the human population is still a major public health threat, as it can lead to devastating pandemics^2^. Influenza A viruses are members of the *Orthomyxoviridae* family characterized by a segmented, negative-sense RNA genome that replicate in the cell nucleus. The eight genomic viral RNA (vRNA) molecules are associated with the nucleoprotein (NP) and with the three subunits of the polymerase complex (PB1, PB2 and PA) to form eight viral ribonucleoproteins (vRNP) that represent the minimal units for transcription and replication^3^.

Genome segmentation confers evolutionary advantages to influenza A viruses by allowing exchange of segments through a process known as genetic reassortment. Viruses responsible for recent pandemics in the 20^th^ and early 21^st^ centuries have been shown to be reassortants between human and animal influenza A viruses, leading to the introduction in humans of new viruses to which the population had essentially no pre-existing immunity^4^. However, genome segmentation also complicates genome packaging and requires particular mechanisms to ensure that a complete set of the eight genomic vRNPs is incorporated into newly formed viral particles. Two models of incorporation have long been debated: a random incorporation model requiring that either less or more than eight vRNPs can be packaged, and a selective incorporation model with packaging of only eight vRNPs, one of each kind^5^,^6^. With growing evidence to support the latter model, a selective mechanism for packaging involving defined segment- and strain-specific packaging signals is now well established. However, it was shown that the majority of viral particles fail to express one or more gene products, suggesting that particles with incomplete genomes might arise^7^,^8^.

Each genomic vRNA is composed of a central coding region, flanked on both sides by non-coding (NC) sequences. The NC sequences are themselves divided into conserved (12 and 13 nucleotides at the extreme 3′ and 5′ ends, respectively) and non-conserved sequences, whose lengths vary between segments^9^,^10^. In vRNPs, the 3′ and 5′ NC regions associate and form secondary structures. The conserved NC sequences were shown to act as a promoter for transcription and replication of the vRNAs^11^,^12^, while the non-conserved NC regions play a role in modulating the efficiency of transcription and replication^13^,^14^. Numerous studies have demonstrated that packaging signals of all eight segments encompass sequences from both the 3′ and 5′ NC regions and from the adjacent coding regions in the vRNA^6^,^15^,^16^,^17^,^18^,^19^,^20^,^21^,^22^. These studies also revealed that mutations in the packaging signals of a given segment could impact the incorporation of other segments. It was therefore proposed that the packaging signals could have two distinct functions: the 3′ and 5′ NC regions of each vRNA would act as incorporation signals to package the vRNP, while the sequences in the coding regions would serve as bundling signals required to pre-assemble the eight different vRNPs prior to their packaging at the budding site^23^. It was suggested that this assembling process is hierarchical^24^,^25^ and that the PB2, PA, NP and M segments play a pivotal role, while the PB1, HA, NA and NS segments are less important^26^.

Gao and Palese evaluated the possibility to exchange packaging signals of the HA segment, with the aim to engineer live attenuated vaccines with a limited risk of potential reassortment^27^. This was successfully achieved by exchanging the complete packaging sequences, *i.e.* signals in the NC and coding sequences. We wondered whether decrease of virus fitness and prevention of reassortment could also be achieved by exchange or replacement of the NC sequences only.

We and others have recently shown that the sequence of the non-conserved NC regions is not critical for efficient rescue of infectious viruses, suggesting the existence of some degree of flexibility in the compatibility between the packaging signals in the NC and coding regions^28^,^29^,^30^. In the present study, our strategy was to produce influenza A viruses that harbour two segments with identical sets of NC regions, while maintaining the presumed bundling signals in the coding regions. We focused on the NA segment of influenza virus A/WSN/33 (WSN) and replaced the NC regions of the NA segment with those of each of the seven other segments. All recombinant viruses were successfully rescued and were characterized by a reduced virus fitness related to a defect in NA segment incorporation. Upon serial passages in cell culture, we found that the length of the heterologous 5′NC region was key to the type of changes required for virus fitness recovery and restoration of incorporation of the NA segment.

## Results

**Figure 1:**
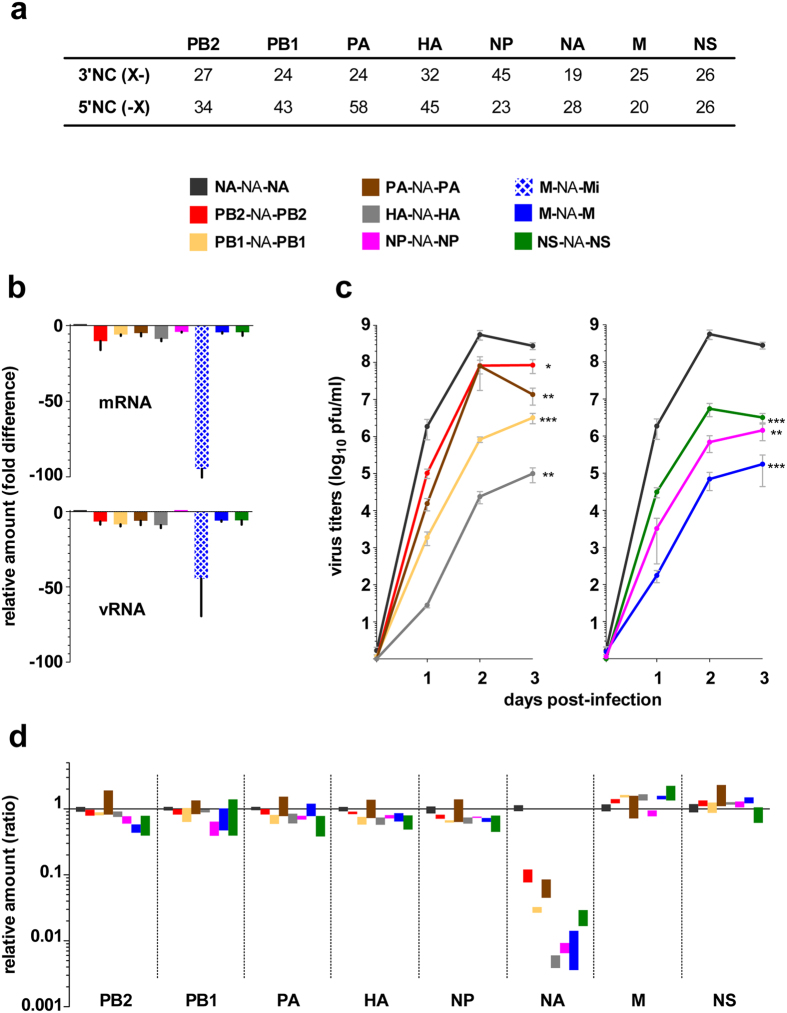
Effects of the replacement of the NA segment NC regions on transcription/replication, growth kinetics and segment incorporation. (**a**) Length, in nucleotides, of the WSN influenza virus 3′ and 5′ NC sequences of each segment (start and stop codons not included). Recombinant **X**-NA-**X** reverse genetics plasmids were constructed and each construct was assigned a colour code. (**b**) Transcription/replication of the **X**-NA-**X** genomic segments evaluated by transient reconstitution of functional vRNPs. 293 T cells were transfected and total RNA was extracted at 24 h post-transfection. The amounts of viral vRNA and mRNA were measured by specific two-step RT-qPCRs^19^. Fold differences relative to **NA**-NA-**NA** were calculated. Three independent experiments were performed and the mean (with Standard Error to the Mean, SEM) is given. (**c**) Growth kinetics of rescued **X**-NA-**X** viruses. Infections were performed in MDCK cells at a low m.o.i. (10^−5^ pfu/cell). Titres were determined by standard plaque assays at the indicated time points. Results correspond to the mean (±SEM) of three independent experiments. Area Under Curve values were compared by t-test with **NA**-NA-**NA** as reference (*0.05 < p-value < 0.01; **0.01 < p-value < 0.001; ***p-value < 0.001). (**d**) Quantification of vRNA levels of the **X**-NA-**X** viruses. Viral RNAs were extracted from the supernatants collected at day three post infection of the growth kinetics in c and the level of each segment was evaluated by specific two-step RT-qPCR^19^. Results were expressed as relative amounts calculated using the 2^−ΔΔCt^ method with **NA**-NA-**NA** as virus reference and segment M as segment reference, except for segment M quantification where segment NP was used as segment reference. Each bar represents the range (minimum - maximum) of the values obtained from three independent growth kinetics.

In the unidirectional reverse genetics plasmid driving the synthesis of segment 6 (NA) vRNA of WSN, sequences of the NC regions were exchanged by those of each of the other segments to generate seven chimeric segment 6 vRNA constructs (Fig. 1a and Supplementary Table S1). Each construct was named **X**-NA-**X**, where **X**- represents the segment origin of the 3′NC region, NA, the neuraminidase coding region including its natural stop codon, and -**X**, the segment origin of the 5′NC region.

### Replacement of segment 6 NC regions by those of other segments did not affect transcription/replication of segment 6 *in vitro*

We first assessed the transcription/replication levels of the different **X**-NA-**X** constructs by transient reconstitution of functional vRNPs. All **X**-NA-**X** segments, with one exception, were transcribed to produce mRNAs and replicated to produce vRNAs by the viral polymerase complex at levels similar to those of the wild-type segment **NA**-NA-**NA** (Fig. 1b). In the case of the replacement with the original M NC regions, *i.e.* following our initial strategy of using the M 5′NC region and the NA stop codon (**M**-NA-**Mi**), transcription was nearly 100-fold lower than for **NA**-NA-**NA**, and replication was also affected (Fig. 1b, hatched blue). The 5′NC region of segment 7 (M) is the shortest one in the WSN genome (Fig. 1a) and the stop codon is merged with the polyU sequence (5′NC: aguagaaacaagguagUUU**UUA**). The use of the cognate NA stop codon in the **M**-NA-**Mi** construct thus shortened the polyU sequence (5′NC: aguagaaacaagguagUUU**CUA**, Supplementary Table S1). Restoration of the M stop codon and thus of a functional polyU (construct **M**-NA-**M**, Supplementary Table S1) led to efficient transcription and replication (Fig. 1b, solid blue). Consequently, construct **M**-NA-**M** was used as basis for all further experiments and related constructs. In agreement with the fact that transcription and replication of the **X**-NA-**X** segments was similar to wild-type, no major differences in expression levels of the NA as analysed by western-blot were observed between the different constructs tested and wild-type, except for **PB1**-NA-**PB1** and **HA**-NA-**HA** for which expression of the NA was reduced (Supplementary Figure S8a).

### Replacement of segment 6 NC regions by those of other segments impaired virus fitness and segment 6 packaging

All chimeric viruses harbouring two identical sets of NC regions could be rescued by reverse genetics and working stocks were constituted at low multiplicity of infection (m.o.i. = 10^−5^) from amplifications of plaque-purified viruses. Sequencing of the whole genome (NC and coding regions of the eight segments) of the working stocks did not reveal any differences with the sequence of the plasmids used for reverse genetics. Viral fitness was assessed by analysis of growth kinetics at low m.o.i. in MDCK cells. Viral growth was significantly impaired for all constructs compared to wild-type. At three days post-infection (p.i.), titres for **PB1**-NA-**PB1**, **HA**-NA-**HA**, **NP**-NA-**NP**, **M**-NA-**M** and **NS**-NA-**NS** were reduced by 2–4 log_10_, and those for **PB2**-NA-**PB2** and **PA**-NA-**PA** were reduced by 1–2 log_10_ compared to wild-type (**NA**-NA-**NA**) (Fig. 1c). Sequencing of the whole genome of the viruses from the clarified supernatants at day 3 post-infection did not reveal any mutation. A significant deficit in the incorporation of the NA segment into virions was observed for the seven chimeric **X**-NA-**X** constructs compared to **NA**-NA-**NA**, when all the other vRNA segments were incorporated with the same efficiency for all viruses (Fig. 1d). Impairment of virus growth was significantly correlated to the deficit of NA segment incorporation (non-parametric correlation test to compare Area Under Curve deduced from the growth curves versus NA segment incorporation fold difference; Spearman r: 0.8682, 95% CI: 0.7660 to 0.9276, two-tailed p-value: < 0.0001). Absolute quantification of the M segment using a synthetic RNA transcript standard curve allowed to estimate the ratio of infectivity, *i.e.* the number of RNA copies per infectious unit (pfu), for the different **X**-NA-**X** viruses. All **X**-NA-**X** viruses produced more (18- to 3000-fold) non-infectious particles than the wild-type **NA**-NA-**NA** virus in accordance with the observed reduction in virus fitness (Table 1).

### Restoration of virus fitness and segment 6 packaging by serial passages

**Figure 2:**
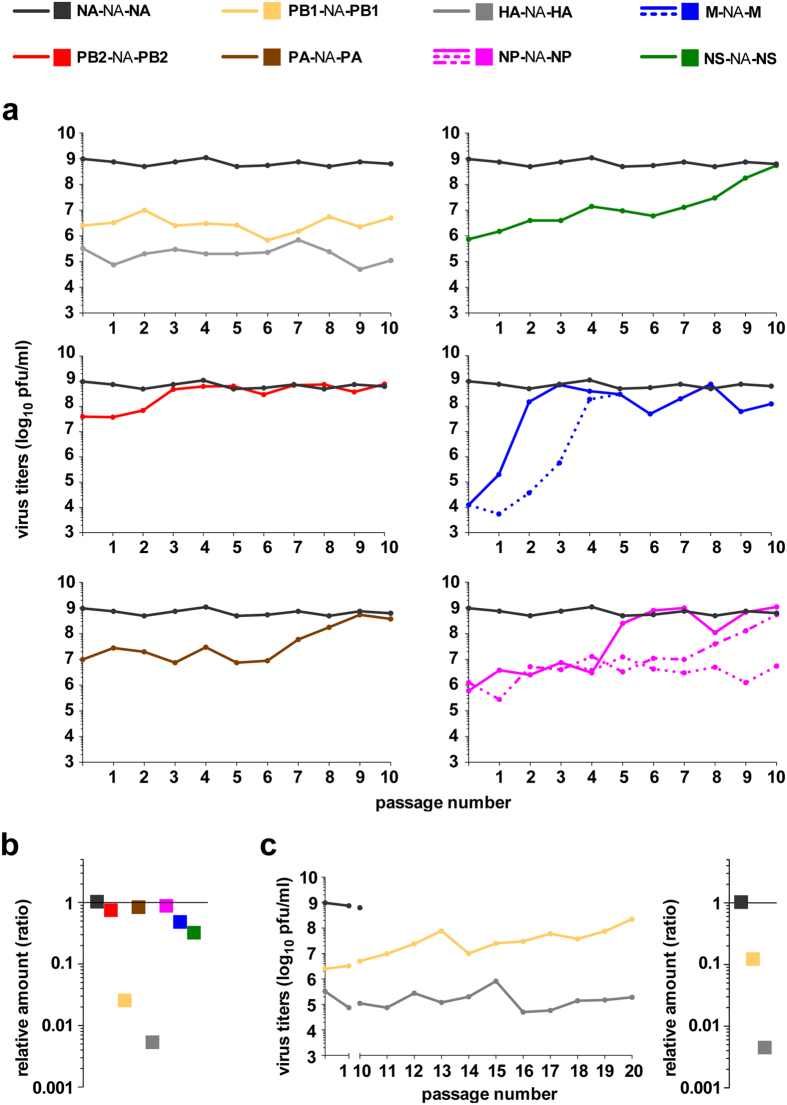
Effects of X-NA-X virus serial passages on virus fitness and segment 6 incorporation. (**a**) **X**-NA-**X** viruses were passaged in MDCK cells at a controlled low m.o.i. (10^−5^ pfu/cell). For each passage, supernatants were collected at day three post-infection, and virus titres were determined. Ten serial passages were performed for each virus. For **M**-NA-**M** and **NP**-NA-**NP**, one or two additional independent serial passages were performed, respectively. (**b**) Incorporation of segment 6 at passage 10. The NA segment vRNA level was evaluated by specific two-step RT-qPCR^19^. Results were expressed as relative amounts calculated using the 2^−ΔΔCt^ method with **NA**-NA-**NA** as virus reference and segment M as segment reference. (**c**) For the **HA**-NA-**HA** and **PB1**-NA-**PB1** viruses, ten additional passages were performed and virus titres determined as in a (left panel). The level of segment 6 incorporation was evaluated at passage 20 as described in b (right panel).

A series of ten passages at controlled, low m.o.i. (10^−5^) was performed in MDCK cells for all chimeric **X**-NA-**X** viruses. For all viruses except **HA**-NA-**HA** and **PB1**-NA-**PB1**, virus titre at passage 10 was similar to that of wild-type **NA**-NA-**NA** (Fig. 2a). For **PB2**-NA-**PB2**, the chimeric virus with the smallest initial growth defect, titres reached wild-type level at passage 3 (Fig. 2a). For **PA**-NA-**PA** and **NS**-NA-**NS** viruses that displayed an initial defect of 2–3 log_10_, restoration of virus fitness was slower, and a titre similar to that of **NA**-NA-**NA** was observed at passages 9 and 10 respectively (Fig. 2a). For **M**-NA-**M** and **NP**-NA-**NP** viruses, that exhibited the strongest initial growth defect, restoration of virus fitness occurred rapidly after two and five passages, respectively (Fig. 2a, solid line). To confirm this rapid restoration of virus fitness, a second, independent series of ten passages was performed for these two viruses. Titres of **M**-NA-**M** virus again rapidly reached wild-type levels (passage 4) (Fig. 2a, dotted line), but **NP**-NA-**NP** titres remained stable and around three log_10_ below that of **NA**-NA-**NA** up to passage 10 (Fig. 2a, dotted line). A third series of ten passages was performed for **NP**-NA-**NP**, leading to a slow increase of virus titres that reached wild-type levels at passage 10 (Fig. 2a, hashed/dotted line).

The level of incorporation of the different segments was evaluated at passage 10 for all **X**-NA-**X** viruses. Restoration of virus fitness was found to be linked to efficient incorporation of the NA segment into virus particles, with similar levels of segment NA detected for all passage 10 viruses except for **HA**-NA-**HA** and **PB1**-NA-**PB1** (Fig. 2b), and to an improved ratio of infectivity (Table 1). As expected, the level of incorporation of the other segments remained similar to that of wild-type for all viruses (Supplementary Fig. S1).

For the **HA**-NA-**HA** and **PB1**-NA-**PB1** viruses, a series of ten additional passages was performed. The titre for **HA**-NA-**HA** consistently remained more than 3 log_10_ below that of wild-type (Fig. 2c), with segment 6 packaging still impaired (Fig. 2c, right panel). On the contrary, for **PB1**-NA-**PB1,** titres increased very slowly during these additional ten passages, almost reaching wild-type levels (Fig. 2c), and the incorporation efficiency of the NA segment also improved, although it was not totally restored (Fig. 2c, right panel). The ratio of infectivity expressed in genomic RNA copies per infectious unit was in agreement with the observed fitness (Table 1).

Using viral RNA extracted from clarified supernatants collected at three days p.i. at the different, indicated passages, sequencing of the whole genome (NC and coding sequences of the eight segments) was performed to identify potential mutations responsible for restoration of virus fitness and segment 6 packaging. Mutations were identified when the viral titre was restored and were traced back at earlier passages. Sanger sequencing sometime revealed double populations at these earlier passages (details are available upon request), but all the identified mutations that were further tested were fixed when virus fitness and segment 6 incorporation were fully restored. These fixed mutations were then reintroduced in the viral genome by site-directed mutagenesis, either alone or in the detected combinations, and corresponding viruses rescued by reverse genetics were tested for viral fitness and segment 6 packaging. We chose to only focus on the viruses for which viral fitness and segment 6 packaging were restored after serial passages. Based on the mutations identified, the **X**-NA-**X** constructs were grouped and are presented according to the length of the -**X** 5′NC region (Fig. 1a): longer than the -NA 5′NC region (**PB1**-NA-**PB1**, **PB2**-NA-**PB2** and **PA**-NA-**PA**) and shorter than the -NA 5′NC region (**NS**-NA-**NS**, **M**-NA-**M** and **NP**-NA-**NP**). The sequences of the different constructs tested are given in Supplementary Table S1. All the substitutions in coding regions are numbered from the start codon in the open reading frame, while substitutions in the NC regions are numbered from their respective extremities.

### Restoration of fitness for X-NA-X viruses with longer 5′NC regions is characterized by point mutations in the NA coding and/or 3′NC regions

For the **PB1**-NA-**PB1** virus, substitutions G17A in the 3′NC region and U1116C and G1359A in the NA coding region were identified at passage 20, but not at earlier passages. In addition, substitution U1572C in the PB1 segment was found from passage 10. Introduction of this latter mutation in the PB1 segment did not alter virus fitness, neither in the **PB1**-NA-**PB1** nor in the **NA**-NA-**NA** context (Supplementary Fig. S2a). Introduction of each of the other three mutations in **PB1**-NA-**PB1** did not improve virus fitness nor segment 6 packaging, but the combination of the three mutations together slightly increased both virus fitness and incorporation of segment 6 (Supplementary Fig. S2b and c). A defect in virus fitness and segment 6 incorporation was also observed when we infected human lung A549 cells with **PB1**-NA-**PB1** (Supplementary Fig. S2d).

**Figure 3:**
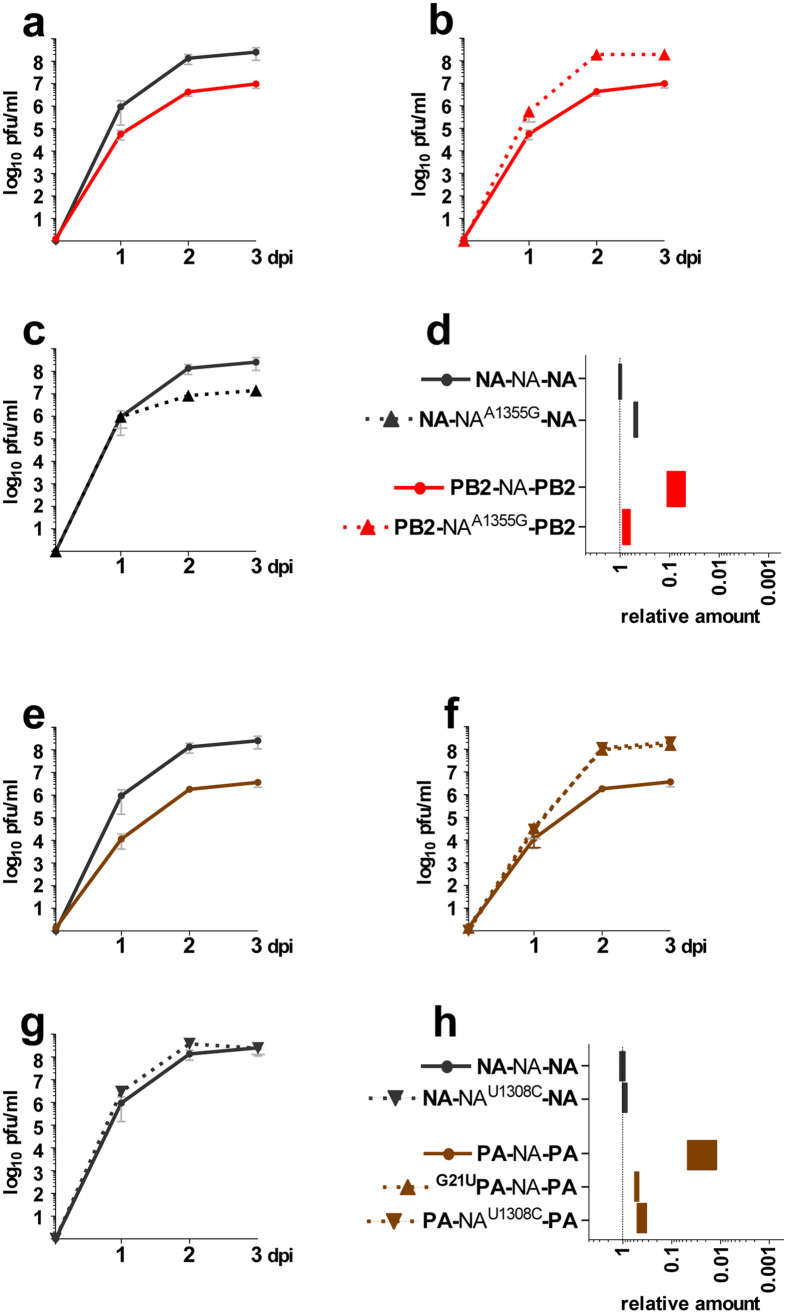
Effects on virus fitness and segment 6 incorporation of mutations acquired by X-NA-X viruses with a longer 5′NC region. Panels (a–d) and (e–h) correspond to **PB2**-NA-**PB2** and **PA**-NA-**PA** viruses and mutants, respectively. (**a**–**c** and **e**–**g**) Growth kinetics. Infections were performed in MDCK cells at a low m.o.i. (10^−5^ pfu/cell). Titres were determined by standard plaque assays at indicated time points. Results correspond to the mean of at least two independent experiments. (**d** and **h**) Incorporation of segment 6. Viral RNAs were extracted from the supernatants collected at three days post infection of the growth kinetics and the NA segment vRNA level was evaluated by specific two-step RT-qPCR^19^. Results were expressed as relative amounts calculated using the 2^−ΔΔCt^ method with **NA**-NA-**NA** as virus reference and segment M as segment reference. Each bar represents the range (minimum - maximum) of the values obtained from the growth kinetics. Symbols used in the growth kinetics graphs are presented next to the name of each virus in panels d and h.

For **PB2**-NA-**PB2,** restoration of virus fitness occurred between passages 2 and 3 (Fig. 2a). Only two substitutions, both non-synonymous, one in the NA coding region (A1355G leading to Asp452Gly) and one in the HA coding region (A1145G leading to Lys382Arg) were identified. The latter substitution in the HA segment, when introduced in both the **PB2**-NA-**PB2** and **NA**-NA-**NA** contexts, did not alter virus fitness nor segment 6 incorporation (Supplementary Fig. S3a and c). On the contrary, the sole introduction of A1355G in **PB2**-NA-**PB2** was sufficient to increase both virus fitness and segment 6 packaging to wild-type levels (Fig. 3a, b and d). Introduction of A1355G in **PB2**-NA-**PB2** also restored wild-type levels of NA expression in MDCK cells infected at a high m.o.i. of 5 pfu/cell (Supplementary Figure S8b). Interestingly, when introduced in the **NA**-NA-**NA** context, mutation A1355G decreased virus fitness to the level of the original **PB2**-NA-**PB2** (Fig. 3c), but segment 6 incorporation was only marginally reduced (Fig. 3d). As assessed by western blot, expression of the NA from the **NA**-NA^A1355G^-**NA** segment was not affected in a transient vRNA reconstitution assay, but NA expression was reduced in MDCK cells infected at a high m.o.i. of 5 pfu/cell in accordance with the decreased virus fitness and segment 6 incorporation (Supplementary Fig. S8a and S8b). When A549 cells were infected, similar results were obtained for **PB2**-NA-**PB2**, but virus fitness of **PB2**-NA^A1355G^-**PB2** seemed to remain affected although segment 6 incorporation was essentially restored as observed in MDCK cells (Supplementary Fig. S3d). Also, the effects of mutation A1355G in the **NA**-NA-**NA** context (**NA**-NA^A1355G^-**NA**) appeared to be exacerbated in A549 as compared to MDCK cells.

Viral titres of **PA**-NA-**PA** increased slowly between passages 6 and 9 (Fig. 2a), but mutations were only detected between passages 7 and 8. Substitutions G21U in the 3′NC region, U1308C in the NA coding region, and G1809A in the PA coding region were found at passage 8, when the titre almost reached wild-type levels, but not at passage 7, when the titre was still one log_10_ lower. Introduction of the PA segment synonymous substitution G1809A in both **PA**-NA-**PA** and **NA**-NA-**NA** contexts did not alter virus fitness nor segment 6 incorporation (Supplementary Fig. S4a and c). Both the single introduction of the G21U substitution in the 3′NC region or of the synonymous U1308C substitution in the NA coding region of **PA**-NA-**PA** allowed the restoration of virus fitness and segment 6 packaging to **NA**-NA-**NA** levels (Fig. 3e, f and h). Either mutation in the **PA**-NA-**PA** context also restored NA expression to wild-type levels in infected MDCK cells (Supplementary Fig. S8b). No additional effect was observed when these two mutations were combined (Supplementary Fig. S4b and c). When introduced into **NA**-NA-**NA,** the U1308C substitution did not affect virus fitness (Fig. 3g) nor incorporation of segment 6 (Fig. 3h), nor the level of NA expression in MDCK cells (Supplementary Fig. S8b). Similar results were obtained for **PA**-NA-**PA**, ^**G21U**^**PA**-NA-**PA**, **PA**-NA^U1308C^-**PA**, **NA**-NA-**NA** and **NA**-NA^U1308C^-**NA** when A549 cells were used (Supplementary Fig. S4d).

### Restoration of fitness for X-NA-X viruses with shorter 5′NC regions is characterized by insertions in the 5′NC sequence

**Figure 4:**
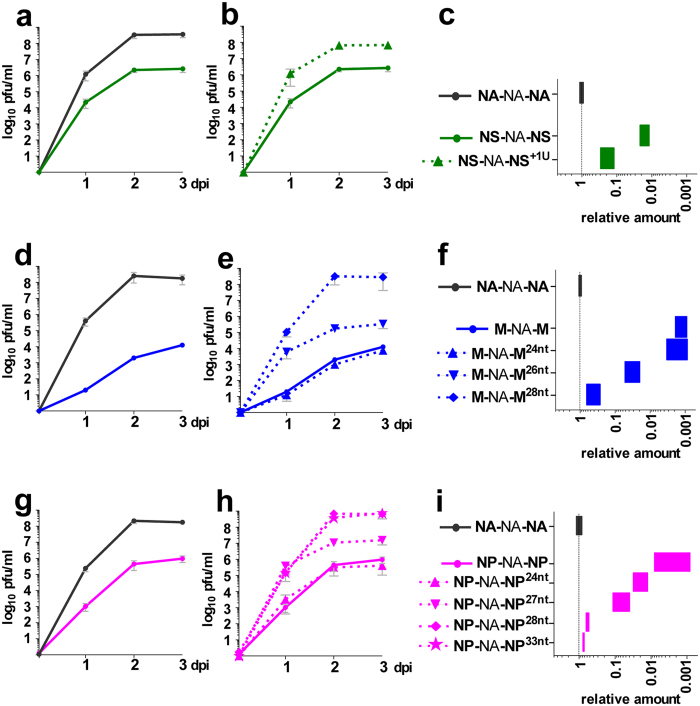
Effects on virus fitness and segment 6 incorporation of mutations acquired by X-NA-X viruses with a shorter 5′NC region. Panels (a–c), (d–f) and (g–i) correspond to **NS**-NA-**NS**, **M**-NA-**M** and **NP**-NA-**NP** viruses and mutants, respectively. (**a**, **b**, **d**, **e**, **g** and **h**) Growth kinetics. Infections were performed in MDCK cells at a low m.o.i. (10^−5^ pfu/cell). Titres were determined by standard plaque assays at indicated time points. Results correspond to the mean of at least two independent experiments. (**c**, **f** and **i**) Incorporation of segment 6. Viral RNAs were extracted from the supernatants collected at three days post infection of the growth kinetics and the NA segment vRNA level was evaluated by specific two-step RT-qPCR^19^. Results were expressed as relative amounts calculated using the 2^−ΔΔCt^ method with **NA**-NA-**NA** as virus reference and segment M as segment reference. Each bar represents the range (minimum - maximum) of the values obtained from the growth kinetics. Symbols used in growth kinetics graphs are presented next to the name of each virus in panels c, f and i.

Fitness of the **NS**-NA-**NS** virus increased progressively with passages, and somewhat more steadily from passage 8 (Fig. 2a). Sequencing at passage 10, when the **NS**-NA-**NS** virus titre reached that of wild-type virus, revealed only two mutations: a one-nucleotide insertion before the stop codon in the 5′NC region and a synonymous G993A substitution in the NA open reading frame. The 5′NC insertion was not present before passage 10, but the G993A substitution appeared as early as passage 3. Introduction of the G993A substitution in both the **NS**-NA-**NS** and **NA**-NA-**NA** contexts did not affect virus fitness nor segment 6 incorporation (Supplementary Fig. S5a and b). In contrast, introduction of the one nt insertion in the 5′NC region of **NS**-NA-**NS** was sufficient to increase virus fitness to near wild-type levels (Fig. 4a and b) and to substantially improve incorporation of segment 6 (Fig. 4c). Similar results were obtained when A549 cells were infected with **NS**-NA-**NS**, **NS**-NA-**NS**^**+1U**^, and **NA**-NA-**NA** (Supplementary Fig. S5c).

The initial defect in **M**-NA-**M** virus growth was rapidly compensated upon serial passages and the virus reached a titre similar to that of wild-type at passages 2 and 4 for our first and second series of passages, respectively (Fig. 2a). In both cases, a similar insertion of eight nucleotides in the 5′NC region was found when viral titres were restored (passages 2 and 4) that was not present at earlier passages. This insertion corresponded to a duplication of a sequence encompassing the polyU (Supplementary Table S1). No mutation in the NA coding region was found, but additional substitutions were detected in other segments: the synonymous A33G substitution in the PB1 segment was found from passage 2 of both series, and the non-synonymous A682C (Asn228His) substitution in the PA segment was only found from passage 3 of the second series. However, introduction of these substitutions in the PB1 and PA segments, alone or in combination, in both **M**-NA-**M** and **NA**-NA-**NA** contexts, had no effect on virus fitness nor segment 6 incorporation (Supplementary Fig. S6a and b). In contrast, introduction of the eight nt insertion in the 5′NC region (**M**-NA-**M**^28nt^) was sufficient to increase both virus fitness and segment 6 incorporation to wild-type levels (Fig. 4d, e and f), which also restored the level of NA expression in infected MDCK cells (Supplementary Fig. S8b). Interestingly, this eight-nucleotide insertion extended the 5′NC region from 20 to 28 nt as found in the NA 5′NC region (Supplementary Table S1 and Fig. 1a), suggesting that the length of the 5′NC region of segment 6 might be important. A chimeric virus with a 24nt-long 5′NC region (**M**-NA-**M**^24nt^) exhibited altered growth properties similar to **M**-NA-**M**, whereas increasing the length of the 5′NC region to 26 nt (**M**-NA-**M**^26nt^) clearly improved both virus fitness and segment 6 incorporation, but to an intermediate level between **M**-NA-**M** and wild-type (Fig. 4d, e and f) that was not sufficient to improve NA expression levels in infected MDCK cells (Supplementary Fig. S8b). Overall, infections of A549 cells with **M**-NA-**M**, **M**-NA-**M**^**26nt**^, **M**-NA-**M**^**28nt**^, and **NA**-NA-**NA**, gave similar results for both virus fitness and segment 6 incorporation, even if the effect appeared to be less pronounced for **M**-NA-**M** (Supplementary Fig. S6c).

For the **NP**-NA-**NP** virus, the initial fitness defect was compensated upon serial passages either rapidly at passage 5 (series 1), or slowly from passage 7 (series 3) or not (series 2) (Fig. 2a). Focusing on series 1 and 3, sequencing performed at passage 10 revealed several mutations that were then tracked at earlier passages. Thus, a C22U substitution in the 5′NC region was found at passages 5 and 10 but not at passages 4 and 9 for series 1 and 3, respectively. This substitution was accompanied by an insertion of 10 and 4 nucleotides, respectively, that also corresponded to a duplication of adjacent sequences and extended the 5′NC region from 23 to 33 or 27 nucleotides, respectively. Finally, several additional mutations were also detected in the NA coding region and in other segments (in PB2, PA), but they differed between series 1 and 3 (Supplementary Table S1). Introduction of these substitutions, alone or in combination, in both the **NP**-NA-**NP** and **NA**-NA-**NA** contexts, had essentially no effect on virus fitness nor on segment 6 incorporation or in some cases even resulted in a further decrease of virus fitness in the **NP**-NA-**NP** context (Supplementary Fig. S7a and c). Likewise, introduction of the C22U substitution in the 5′NC region of **NP**-NA-**NP** did not improve but rather decreased virus fitness (Supplementary Fig. S7b and c). Only the insertions in the 5′NC region partially (**NP**-NA-**NP**^27nt^) or totally (**NP**-NA-**NP**^33nt^) restored virus fitness and segment 6 packaging to wild-type levels (Fig. 4g, h and i). Accordingly, the NA expression level remained low (**NP**-NA-**NP**^27nt^) or was restored (**NP**-NA-**NP**^33nt^) in infected MDCK cells (Supplementary Fig. S8b). Like for **M**-NA-**M**, the length of the 5′NC region seemed important and additional constructs were tested (Supplementary Table S1). As observed with **M**-NA-**M** constructs, a chimeric virus with a 24nt-long 5′NC region (**NP**-NA-**NP**^24nt^) also showed reduced fitness and defective incorporation of segment 6, whereas a virus with a 28nt-long 5′NC region (**NP**-NA-**NP**^28nt^) as in wild-type **NA**-NA-**NA** exhibited completely restored virus fitness and segment 6 incorporation levels (Fig. 4g, h and i). Overall, similar results were obtained with **NP**-NA-**NP**, **NP**-NA-**NP**^**27nt**^, **NP**-NA-**NP**^**33nt**^, and **NA**-NA-**NA** when infections of A549 cells were performed (Supplementary Fig. S7d).

## Discussion

The respective role of the packaging signals found in the NC and coding regions is not yet fully understood. It was proposed that the NC signals drive the incorporation of the vRNAs into newly formed viral particles, while sequences in the coding regions serve as a bundling signal to ensure that a complete set of the different genomic vRNPs is packaged^6^,^23^. The requirement for compatibility between the packaging signals in the NC and coding regions has not been studied extensively. Attempts to replace the H1 non conserved NC regions of the HA segment of WSN by those of other HA subtypes resulted in infectious virus production with H2, H3, H5 and H9 NC regions, but not with the shorter H4, H6 or H7 NC regions, when both the 3′ and 5′ ends were replaced. In addition, all chimeric viruses harbouring only one replaced end could be rescued, and the differences observed in viral fitness led the authors to conclude that 1) the 3′ NC region was more important than the 5′NC region, 2) its length impacted viral fitness, and 3) there was no restriction of compatibility between the 3′ and 5′ ends of the HA segment for the different HA subtypes studied^31^. However, the compatibility between the NC and coding regions was not discussed. Our study aimed at evaluating more specifically this compatibility. To this end, the NC regions of the NA segment were replaced by those of the other segments, while maintaining the signals in the coding region. All our **X**-NA-**X** viruses could be rescued and were infectious, even if their fitness was more or less altered. This showed that 1) there is no strict necessity for compatibility between the packaging signals in the NC and coding regions, and 2) influenza A viruses harbouring two genomic segments with identical ends restricted to the NC regions can be infectious, at least when segment 6 is used to carry the duplicate NC regions. However, the compatibility might be more stringent for other segments. Strikingly, for **HA**-NA-**HA**, no restoration of fitness could be observed even after 20 serial passages. When we swapped the NC regions of segment 4 (HA) and 6 (NA), we were unable to recover a **NA**-HA-**NA** + **HA**-NA-**HA** infectious virus (data not shown), suggesting that the packaging signals in the NA NC regions were incompatible with those in the HA coding region or that replacement of specific nucleotides in the HA NC region that were shown to be important by Zhao *et al*.^31^ could actually play a critical role in the specific packaging of the HA segment.

Various studies have shown that mutations introduced in the packaging signals in the coding region of certain segments could affect both the incorporation of that specific segment and that of the other segments^16^,^19^,^20^,^25^,^32^. In our study, only the incorporation of the modified segment 6 was affected when the NC regions were replaced with those of another segment. Thus, by only modifying the NC regions, we did not disturb the incorporation of the other segments and the signals in the coding regions required for the selection of a set of unique genomic segments were preserved. Segment 6 incorporation was nonetheless clearly impaired for all initial **X**-NA-**X** constructs, and this also seemed to have consequences on virus fitness. However, segment 6 is not considered as a major actor in influenza A virus genome assembling and packaging^25^,^26^, and viruses lacking segment 6 have previously been obtained^7^,^33^ and even observed in nature^34^. Parallel to their defect in segment 6 incorporation, all the initial **X**-NA-**X** viruses produced far more non-infectious particles than the wild-type **NA**-NA-**NA** virus, but ratios of infectivity were also improved with segment 6 incorporation (Table 1). Transient functional vRNP reconstitution assays showed that transcription into mRNA was not affected for the **X**-NA-**X** segments, and neither was NA protein expression except for **PB1**-NA-**PB1** and **HA**-NA-**HA** (Fig. 1b and Supplementary Fig. S8a). However, in infected cells, the levels of NA protein expression by the different **X**-NA-**X** viruses tested essentially reflect the level of segment 6 incorporation (Supplementary Fig. S8b). For the **PB1**-NA-**PB1** and **HA**-NA-**HA** segments, NA protein expression (Supplementary Fig. S8a), but not NA mRNA production (Fig. 1b), was clearly reduced in functional vRNP transient reconstitution assays, suggesting a defect at the level of translation. Since all the **X**-NA-**X** viruses could be rescued by reverse genetics, what causes the specific reduction in NA protein expression remains to be investigated for these two constructs.

Sequences in the coding regions acting as bundling signals to bring together the different genomic vRNPs are believed to function via direct RNA-RNA interactions^35^,^36^ or via indirect interactions mediated by a yet unknown viral or cellular protein^17^. Until now, no putative interacting motifs could be identified using *in silico* analyses, most probably because 1) viral RNA coils around the NP proteins in a complex way in the vRNPs, 2) packaging signals could potentially be discontinuous, and 3) RNA-RNA interactions might involve non Watson-Crick base-pairing^17^. Direct RNA-RNA interactions have been demonstrated *in vitro*^35^,^36^,^37^,^38^ and the networks of interaction between the eight vRNAs that were obtained for different subtypes of influenza A viruses highlighted the complexity of the bundling process and how difficult it might be to identify the specific nucleotides involved. Single, synonymous mutations at extremely conserved positions within the coding region were nonetheless shown to be sufficient to impair the incorporation of the specific segment into viral particles^20^,^39^. Our approach based on serial passaging of the chimeric viruses was successful in identifying specific residues in the NA coding regions that could be important for interactions with other segments. Mutations A1355G in **PB2**-NA-**PB2** and U1308C in **PA**-NA-**PA** appeared near the 5′NC region and were shown to restore segment 6 incorporation and virus fitness. Since both the PB2 and PA 5′NC regions were longer than the original 5′NC region of segment 6, our results suggest that these mutations restored the necessary links between segment 6 and another segment to allow its efficient packaging. Introduction of these mutations into wild-type **NA**-NA-**NA** had different consequences. Mutation A1355G (found in the passaged **PB2**-NA-**PB2)** impaired to some extent virus fitness (Fig. 3c) and segment 6 incorporation (Fig. 3d) in MDCK cells. The effect was more pronounced in A549 cells (Supplementary Fig. S3d). This suggests a putative, additional role of the amino-acid change resulting of the A1355G mutation, although this deleterious effect was not obviously seen for the **PB2**-NA^A1355G^-**PB2** virus infection in A549 cells (Supplementary Fig. S3d). In addition, the effect on virus fitness and segment 6 incorporation in infected MDCK was shown to be base dependent in **PB2**-NA-**PB2** (Supplementary Fig. S3b and c). As for mutation U1308C (found in the passaged **PA**-NA-**PA)**, its introduction in **NA**-NA-**NA** had no effect (Fig. 3g). We therefore hypothesized that the specific distance of the mutated nucleotide from one of the ends might be the key determinant. However, our various attempts at clarifying this mechanism by introducing different substitutions in both **NA**-NA-**NA** and **PA**-NA-**PA** did not lead to any conclusive results (data not shown), and therefore how mutation U1308C affects segment 6 incorporation remains to be determined.

When the 5′NC region of segment 6 was replaced by a shorter 5′NC region, restoration of virus fitness always involved insertions in the 5′NC region, either as a point insertion (**NS**-NA-**NS**) or as a partial duplication (**M**-NA-**M** and **NP**-NA-**NP**). Insertion of random sequences revealed that the nucleotide sequence of the insertion was not important as such (data not shown). Similarly, it was previously shown that the length of the packaging signals in the coding region near the 5′NC region was more important for segment 7 incorporation than the nucleotide sequence itself^22^. We can conclude from our results in the **M**-NA-**M** and **NP**-NA-**NP** contexts that a minimum length of the 5′NC region is required for efficient packaging of segment 6 and thus for optimal virus fitness. This was further demonstrated by shortening the length of the 5′NC region of the wild-type **NA**-NA-**NA** segment. Segment 6 incorporation was slightly reduced when the 5′NC region was less than 26 nucleotides long (Supplementary Fig. S9) with expected consequences on the level of NA expression in infected MDCK cells (Supplementary Fig. S8b). Results obtained in A549 cells were relatively similar. These observations suggest that the insertions in the 5′NC region that restore the virus fitness of our **X**-NA-**X** constructs allowed the repositioning of essential residues of the coding region involved in vRNP bundling and hence restored efficient packaging of segment 6.

Most of the observed modifications that restored viral fitness occurred in the 5′ region of the vRNA, when others have concluded that the 3′ region might have more impact on viral fitness and segment incorporation^6^,^31^. Even if our results might seem in contradiction, one might argue either that our approach revealed that the 5′ region contains some particularly important signals that have to be restored, or, on the contrary, that our results support the importance of the 3′ region, within which mutations cannot be tolerated.

In conclusion, our results highlight the importance of the signals present in the NA coding region for incorporation of segment 6 into virions. The position of these signals within the NA segment seems to be crucial to assemble the set of eight genomic vRNPs to be packaged by maintaining a correct network of interactions. These results were based only on segment 6 experiments and it would now be necessary to apply a similar approach to other genomic segments. This would help determine, for the other viral segments, whether altering the 5′NC region length could also lead to mutations restoring the positioning of functional bundling signals in the coding regions. Complementary approaches will also be needed to further identify the specific residues involved in the complex network of interactions required for efficient packaging of the influenza virus vRNPs.

## Methods

### Plasmids and reverse genetics

The A/WSN/33 influenza virus 12-plasmid-based reverse genetics system was adapted from previously described procedures^40^,^41^. To construct the **X**-NA-**X** plasmids, the whole NA coding sequence was amplified by PCR using primers containing the entire 3′ or 5′ NC sequences of each segment, including the TAG stop codon of the NA segment (except for the **M**-NA-**M** plasmid which used the TAA stop codon), and the resulting amplicon was cloned into the pPR plasmid as described previously^42^. Point mutations in the coding or NC regions were introduced by site-directed mutagenesis using Quikchange II Site-Directed mutagenesis kit (Agilent) according to the manufacturer’s instructions. In the coding region, numbering of the mutation positions started from the ATG codon. The sequences of the primers used are available upon request. All constructs were verified by Sanger sequencing (Eurofins Genomics).

### Transient reconstitution of functional vRNPs

The four expression plasmids for the A/Puerto Rico/8/34 (PR8) polymerase and NP proteins (a gift from G. Brownlee) were co-transfected in 293 T cells with each of the **X**-NA-**X** constructs using FUGENE HD (Promega). Twenty four hours post-transfection, total RNAs were extracted using the Nucleospin RNA kit (Macherey-Nagel) as described by the manufacturer’s recommendations and reverse transcribed using the 3′ universal primer for the vRNAs (5′AGGGCTCTTCGGCCAGCRAAAGCAGG)^19^ or an oligo dT for the mRNAs. The cDNAs were then used as templates for quantitative PCR (qPCR) using a LightCycler 480 (Roche). Reaction conditions and primers for the detection of the NA segment were essentially as described by Marsh *et al*.^19^. Fold differences were calculated in comparison to the wild-type NA construct using the qPCR theoretical relationship between Crossing threshold (Ct) value differences and PCR product amount variations (a 1 Ct difference means a 2-fold difference in PCR product amount; a 10-fold difference in PCR product amount corresponds to a 3.3 Ct difference). Absolute quantification of the M-segment was performed using an in-house RT-qPCR and a quantified synthetic RNA transcript.

### Cells, virus cloning, amplification and multi-cycle growth kinetics

Madin-Darby canine kidney (MDCK), and human embryonic kidney 293 T and human lung A549 cell cultures were maintained in DMEM supplemented with 5% and 10% foetal calf serum (FCS), respectively. All cells were grown at 37 °C with 5% CO_2_.

Viruses rescued by reverse genetics were titrated and plaque-purified by plaque assay using Avicel^43^ and agarose^44^ overlays, respectively. Plaque-purified viruses were then amplified once in MDCK cells in DMEM supplemented with 1 μg/ml of TPCK-trypsin to constitute working stocks. Virus growth kinetics were performed at an m.o.i. of 10^−5^ in MDCK cells and 10^−4^ in A549 cells. Viral titres in clarified supernatants were determined at the different indicated time points post infection by plaque assay.

### Sequencing of the genomic RNA segments

The sequence of all genomic RNA segments was checked for all rescued viruses. Viral RNA was extracted from clarified supernatant using QIAamp Viral RNA Mini kit (Qiagen). Sequencing of the full-length segment was performed in two steps by sequencing the coding region on the one hand and the NC regions on the other hand. Sequences of the 3′ and 5′ NC regions were amplified by RT-PCR as previously described^45^. After purification, all PCR products were sequenced using the Sanger method (Eurofins Genomics). All primer sequences are available upon request.

### Incorporation of viral RNA segments into virions

Three days post-infection, viral RNA was extracted from infected cell supernatants. Reverse transcription specific for vRNAs and qPCRs were performed as described above. The qPCRs were performed with segment-specific primers using a LightCycler 480 (Roche) as described^19^. The relative amounts of each vRNA were calculated using the 2^−ΔΔCt^ method with wild-type **NA**-NA-**NA** as virus reference and segment M as segment reference, except for segment M quantification where segment NP was used as segment reference.

### Western-blot

Twenty-four hours after transfection or eight hours after infection at high m.o.i. or 24 hours after infection at low m.o.i., cells were solubilised in LDS sample buffer (Invitrogen) supplemented with beta-mercaptoethanol. Proteins were separated by polyacrylamide gel electrophoresis (NUPAGE, Invitrogen), followed by western blotting. Rabbit anti-influenza A virus NS1 polyclonal antibody was a kind gift from D. Marc (INRA-Nouzilly, France). Rabbit anti-influenza A virus NA polyclonal antibody was purchased from GeneTex. Anti-beta-actin (Abcam) and anti-GAPDH (ThermoScientific) were used for cellular controls.

## Additional Information

**How to cite this article**: Crescenzo-Chaigne, B. *et al*. Incorporation of the influenza A virus NA segment into virions does not require cognate non-coding sequences. *Sci. Rep.* **7**, 43462; doi: 10.1038/srep43462 (2017).

**Publisher's note:** Springer Nature remains neutral with regard to jurisdictional claims in published maps and institutional affiliations.

## Supplementary Material

Supplementary Data

## Figures and Tables

**Table 1:** Ratio of infectivity expressed as number of genomic vRNA copies per infectious unit (copies/pfu).

X-NA-X	Viral stock	Virus at passage 10	Virus at passage 20
**NA**-NA-**NA**	10	20	
**PB2**-NA-**PB2**	180	10	
**PB1**-NA-**PB1**	900	500	20
**PA**-NA-**PA**	600	20	
**HA**-NA-**HA**	2000	2000	1400
**NP**-NA-**NP**	3000	10	
**M**-NA-**M**	30000	80	
**NS**-NA-**NS**	7000	20	
**PB2**-NA^A1355G^-**PB2**	16		
**NA**-NA^A1355G^-**NA**	250		
**PA**-NA^U1308C^-**PA**	39		
^**G21U**^**PA**-NA-**PA**	30		
**NA**-NA^U1308C^-**NA**	16		
**M**-NA-**M**^**28nt**^	170		
**M**-NA-**M**^**26nt**^	18000		
**NP**-NA-**NP**^**33nt**^	20		
**NP**-NA-**NP**^**27nt**^	900		
**NS**-NA-**NS**^**+1U**^	60		
**NA**-NA-**NA**^**20nt**^	110
