# Supplementary material for: Incorporation of the influenza A virus NA segment into virions does not require cognate non-coding sequences
Source: Sci Rep. 2017 Feb 27;7:43462. doi: 10.1038/srep43462 (PMC5327478; doi:10.1038/srep43462)
Supplement: Supplementary Data [file srep43462-s1.pdf]

Incorporation of the influenza A virus NA segment into virions does not require cognate non-coding sequences.

Bernadette Crescenzo-Chaigne [1,2,3+], Cyril V.S. Barbezange [1,2,3+#], Stéphane Léandri [1,2,3], Camille Roquin [1,2,3], Camille Berthault [1,2,3] and Sylvie van der Werf [1,2,3*]

[1] Institut Pasteur, Unité de Génétique Moléculaire des Virus à ARN, Paris, France

[2] Unité Mixte de Recherche 3569, Centre National de la Recherche Scientifique, Paris, France

[3] Université Paris-Diderot Sorbonne-Paris-Cité, Paris, France

Supplementary figure legends

Supplementary Figure S1. Incorporation of the different X-NA-X virus segments. (a) Incorporation of the eight segments at passage 10. (b) For **PB1**-NA-**PB1** and **HA**-NA-**HA** viruses, incorporation of the eight segments at passage 20. For (a) and (b), viral RNAs were extracted from the supernatants collected at day three post infection and the level of each segment was evaluated by specific two-step RT-qPCR [19]. Results were expressed as relative amounts calculated using the $2^{-\Delta\Delta Ct}$ method with **NA**-NA-**NA** as virus reference and segment M as segment reference, except for segment M quantification where segment NP was used as segment reference. Results for segment NA are already presented in Fig. 2.

Supplementary Figure S2. Effects of mutations identified in PB1-NA-PB1 virus on virus fitness and segment 6 incorporation. (a-b) Growth kinetics. Infections were performed in MDCK cells at a low m.o.i. (10^{-5} pfu/cell). Titres were determined by standard plaque assays at indicated time points. Results correspond to the mean of at least two independent experiments. (c) Incorporation of segment 6. Viral RNAs were extracted from the supernatants collected at three days post infection of the growth kinetics and the NA segment vRNA level was evaluated by specific two-step RT-qPCR [19]. Results were expressed as relative amounts calculated using the $2^{-\Delta\Delta Ct}$ method with **NA**-NA-**NA** as virus reference and segment M as segment reference. Each bar represents the range (minimum - maximum) of the values obtained from the growth kinetics. Symbols used in the growth kinetics graphs are presented next to the name of each virus in panel c. (d) Infections in A549 cells at a low m.o.i. (10^{-4} pfu/cell). Supernatants were collected at three days p.i. Left panel: viral titres. Right panel: segment 6 incorporation.

Supplementary Figure S3. Effects of additional mutations identified in PB2-NA-PB2 virus on virus fitness and segment 6 incorporation. (a-b) Growth kinetics. Infections were performed

in MDCK cells at a low m.o.i. (10^{-5} pfu/cell). Titres were determined by standard plaque assays at indicated time points. Results correspond to the mean of at least two independent experiments. (c) Incorporation of segment 6. Viral RNAs were extracted from the supernatants collected at three days post infection of the growth kinetics and the NA segment vRNA level was evaluated by specific two-step RT-qPCR [19]. Results were expressed as relative amounts calculated using the $2^{-\Delta\Delta Ct}$ method with **NA**-NA-**NA** as virus reference and segment M as segment reference. Each bar represents the range (minimum - maximum) of the values obtained from the growth kinetics. Symbols used in the growth kinetics graphs are presented next to the name of each virus in panel c. (d) Infections in A549 cells at a low m.o.i. (10^{-4} pfu/cell). Supernatants were collected at three days p.i. Left panel: viral titres. Right panel: segment 6 incorporation.

Supplementary Figure S4. Effects of additional mutations identified in PA-NA-PA virus on virus fitness and segment 6 incorporation. (a-b) Growth kinetics. Infections were performed in MDCK cells at a low m.o.i. (10^{-5} pfu/cell). Titres were determined by standard plaque assays at indicated time points. Results correspond to the mean of at least two independent experiments. (c) Incorporation of segment 6. Viral RNAs were extracted from the supernatants collected at three days post infection of the growth kinetics and the NA segment vRNA level was evaluated by specific two-step RT-qPCR [19]. Results were expressed as relative amounts calculated using the $2^{-\Delta\Delta Ct}$ method with **NA**-NA-**NA** as virus reference and segment M as segment reference. Each bar represents the range (minimum - maximum) of the values obtained from the growth kinetics. Symbols used in the growth kinetics graphs are presented next to the name of each virus in panel c. (d) Infections in A549 cells at a low m.o.i. (10^{-4} pfu/cell). Supernatants were collected at three days p.i. Left panel: viral titres. Right panel: segment 6 incorporation.

Supplementary Figure S5. Effects of additional mutations identified in NS-NA-NS virus on virus fitness and segment 6 incorporation. (a) Growth kinetics. Infections were performed in MDCK cells at a low m.o.i. (10^{-5} pfu/cell). Titres were determined by standard plaque assays at indicated time points. Results correspond to the mean of at least two independent experiments. (b) Incorporation of segment 6. Viral RNAs were extracted from the supernatants collected at three days post infection of the growth kinetics and the NA segment vRNA level was evaluated by specific two-step RT-qPCR [19]. Results were expressed as relative amounts calculated using the $2^{-\Delta\Delta Ct}$ method with **NA**-NA-**NA** as virus reference and segment M as segment reference. Each bar represents the range (minimum - maximum) of the values obtained from the growth kinetics. Symbols used in panel a are presented next to the name of each virus in panel b. (c) Infections in A549 cells at a low m.o.i. (10^{-4} pfu/cell). Supernatants were collected at three days p.i. Left panel: viral titres. Right panel: segment 6 incorporation.

Supplementary Figure S6. Effects of additional mutations identified in M-NA-M virus on virus fitness and segment 6 incorporation. (a) Growth kinetics. Infections were performed in MDCK cells at a low m.o.i. (10^{-5} pfu/cell). Titres were determined by standard plaque assays at indicated time points. Results correspond to the mean of at least two independent experiments. (b) Incorporation of segment 6. Viral RNAs were extracted from the supernatants collected at three days post infection of the growth kinetics and the NA segment vRNA level was evaluated by specific two-step RT-qPCR [19]. Results were expressed as relative amounts calculated using the $2^{-\Delta\Delta Ct}$ method with **NA**-NA-**NA** as virus reference and segment M as segment reference. Each bar represents the range (minimum - maximum) of the values obtained from the growth kinetics. Symbols used in the growth kinetics graphs are presented next to the name of each virus in panel b. (c) Infections in A549 cells at a low m.o.i. (10^{-4} pfu/cell). Supernatants were collected at three days p.i. Left panel: viral titres. Right panel: segment 6 incorporation.

Supplementary Figure S7. Effects of additional mutations identified in NP-NA-NP virus on virus fitness and segment 6 incorporation. (a-b) Growth kinetics. Infections were performed in MDCK cells at a low m.o.i. (10^{-5} pfu/cell). Titres were determined by standard plaque assays at indicated time points. Results correspond to the mean of at least two independent experiments. (c) Incorporation of segment 6. Viral RNAs were extracted from the supernatants collected at three days post infection of the growth kinetics and the NA segment vRNA level was evaluated by specific two-step RT-qPCR [19]. Results were expressed as relative amounts calculated using the $2^{-\Delta\Delta Ct}$ method with **NA**-NA-**NA** as virus reference and segment M as segment reference. Each bar represents the range (minimum - maximum) of the values obtained from the growth kinetics. Symbols used in the growth kinetics graphs are presented next to the name of each virus in panel c. (d) Infections in A549 cells at a low m.o.i. (10^{-4} pfu/cell). Supernatants were collected at three days p.i. Left panel: viral titres. Right panel: segment 6 incorporation.

Supplementary Figure S8. NA protein expression level evavuated by Western-Blot using chemiluminescence acquisition with a G. Box (Syngene). (a) Original and additional mutant constructs tested by functional vRNP transient reconstitution assays. Twenty-four hours after transfection, 293T cell lysates were analysed for viral NA protein and for GAPDH as cellular control. (b) MDCK infections were performed with viruses rescued by reverse genetics. Upper part of b: **PB2**-NA-**PB2** and **PA**-NA-**PA** constructs, as well as **NA**-NA-**NA**, were tested at high m.o.i. (5 pfu/cell) and cell lysates were analysed at eight hours after infection for viral NA and NS1 proteins and for β-actin as cellular control. Lower part of b: **M**-NA-**M**, **NP**-NA-**NP**, and **NA**-NA-**NA** constructs were tested at low m.o.i. (10^{-2} pfu/cell) and cell lysates were analysed at 24 hours after infection for viral NA and NS1 proteins and for β-actin as cellular control.

Supplementary Figure S9. Effects of a shorter 5'NC region in NA-NA-NA virus on virus fitness and segment 6 incorporation. (a) Growth kinetics. Infections were performed in MDCK cells at a low m.o.i. (10^{-5} pfu/cell). Titres were determined by standard plaque assays at indicated time points. Results correspond to the mean of at least two independent experiments. (b) Incorporation of segment 6. Viral RNAs were extracted from the supernatants collected at three days post infection of the growth kinetics and the NA segment vRNA level was evaluated by specific two-step RT-qPCR [19]. Results were expressed as relative amounts calculated using the $2^{-\Delta\Delta Ct}$ method with **NA**-NA-**NA** as virus reference and segment M as segment reference. Each bar represents the range (minimum - maximum) of the values obtained from the growth kinetics. Symbols used in the growth kinetics graphs are presented next to the name of each virus in panel b. The stop codon of the **NA**-NA-**NA**$^{\mathbf{20nt}}$ was modified to respect the polyU sequence (see Supplementary Table S1). (c) Infections in A549 cells at a low m.o.i. (10^{-4} pfu/cell). Supernatants were collected at three days p.i. Left panel: viral titres. Right panel: segment 6 incorporation.

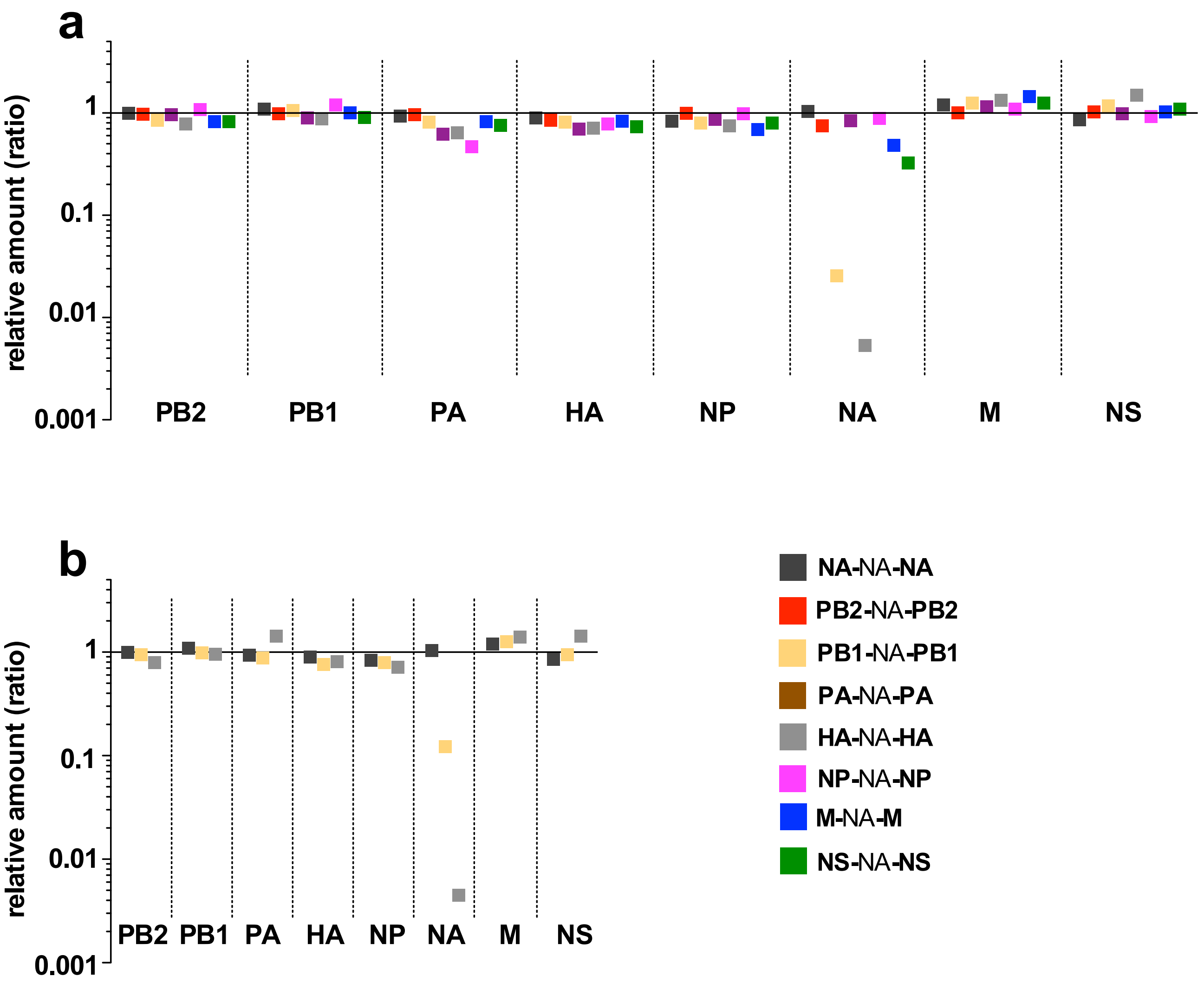

Supplementary Figure S1.

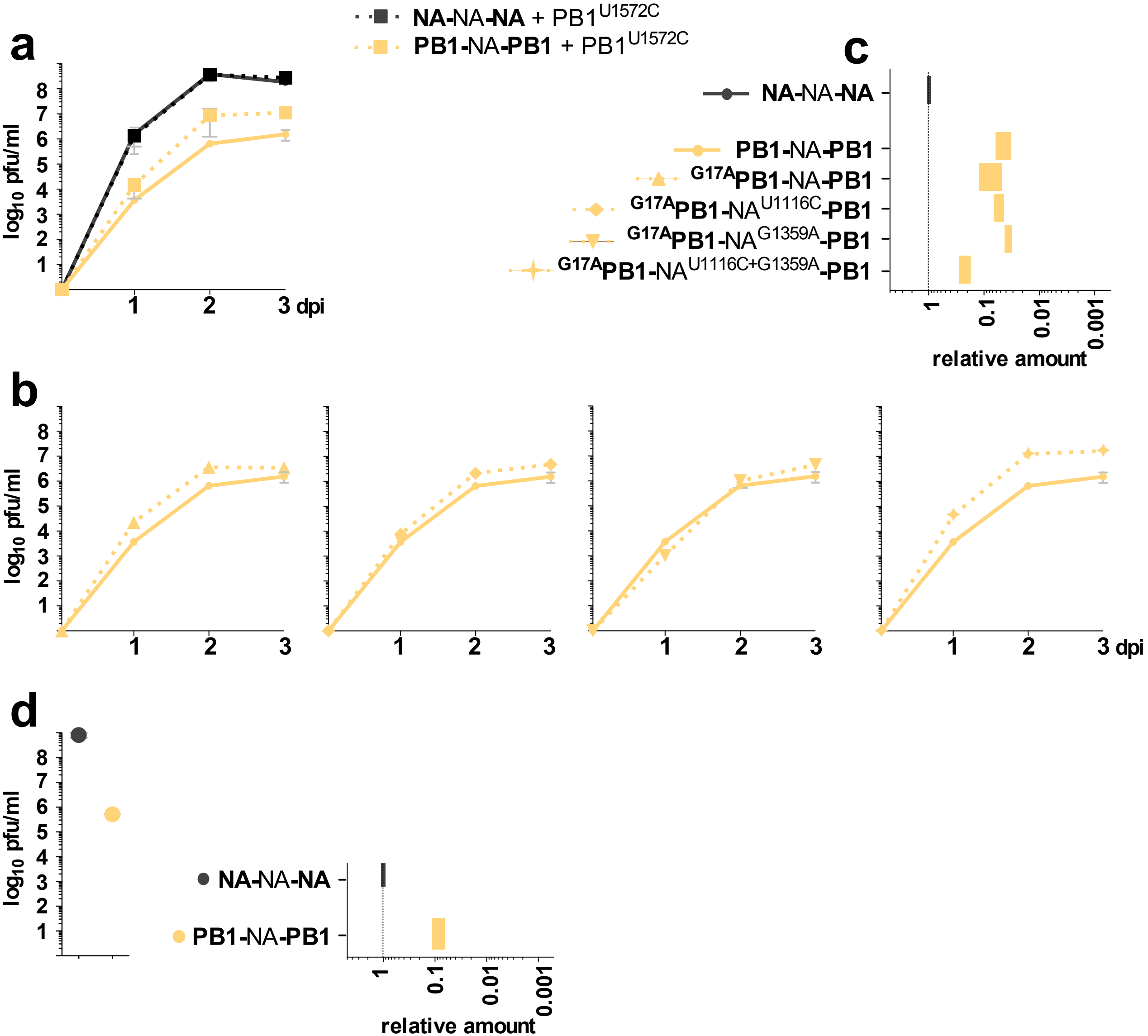

Supplementary Figure S2.

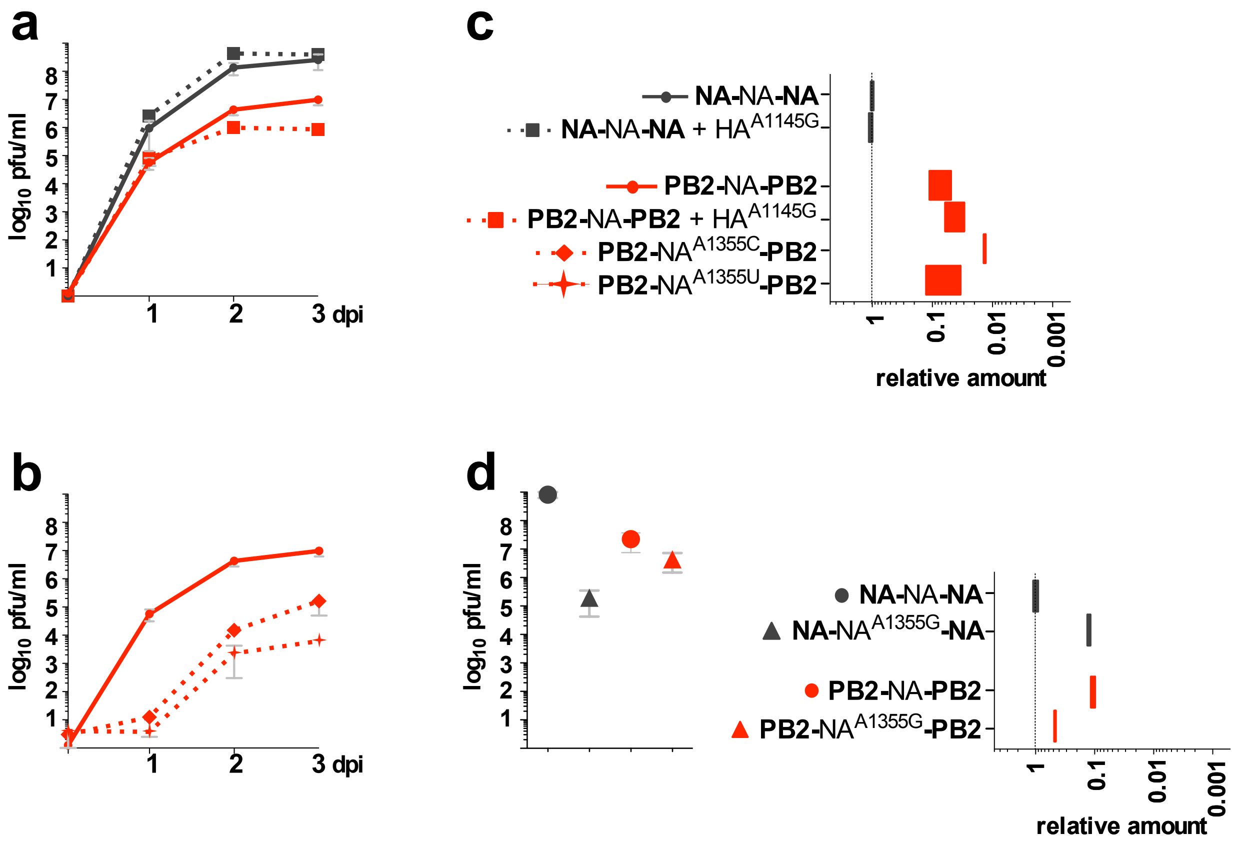

Supplementary Figure S3.

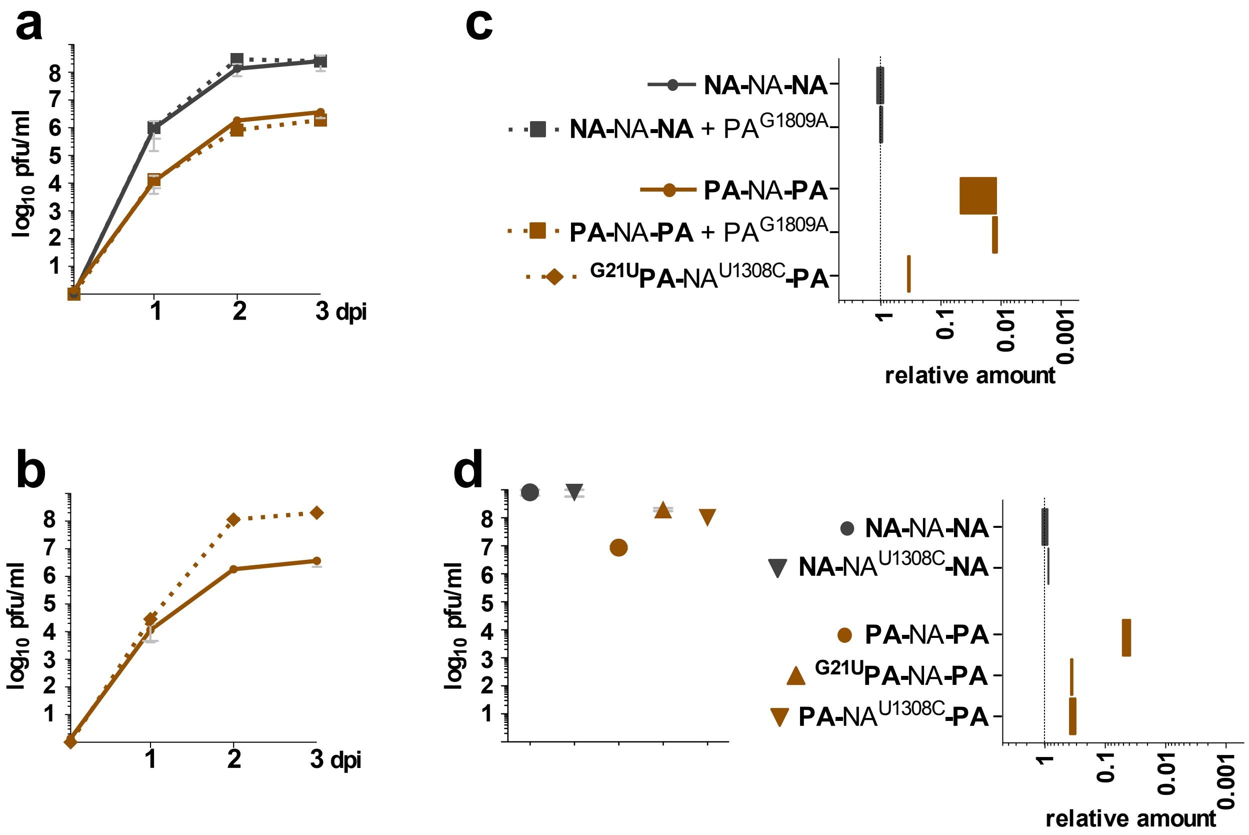

Supplementary Figure S4.

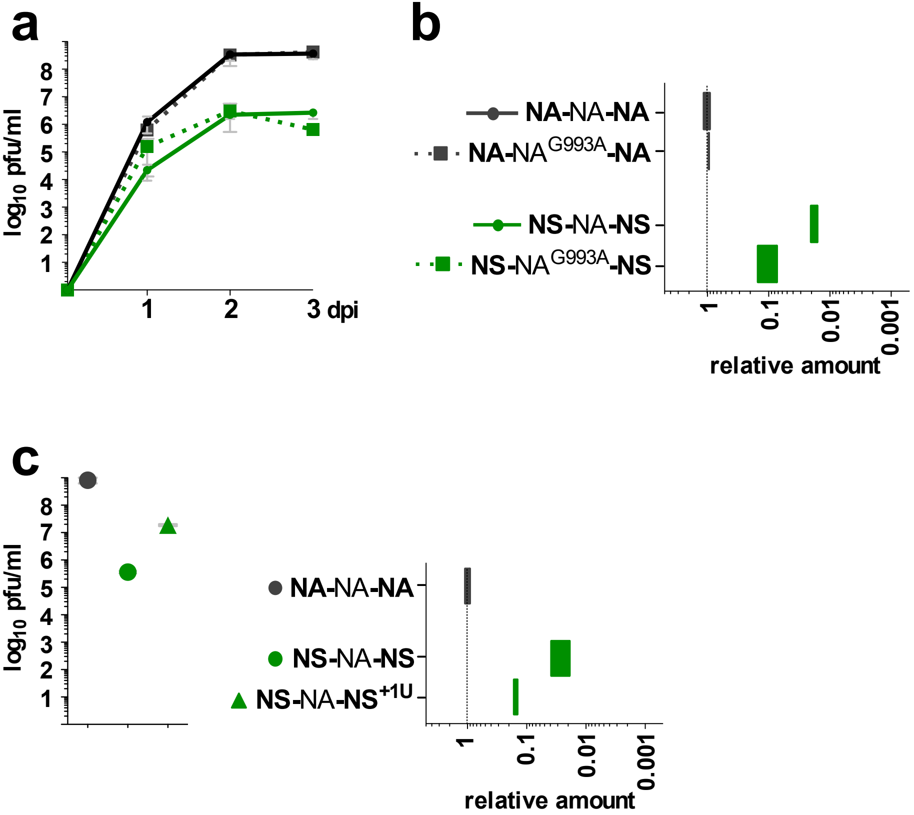

Supplementary Figure S5.

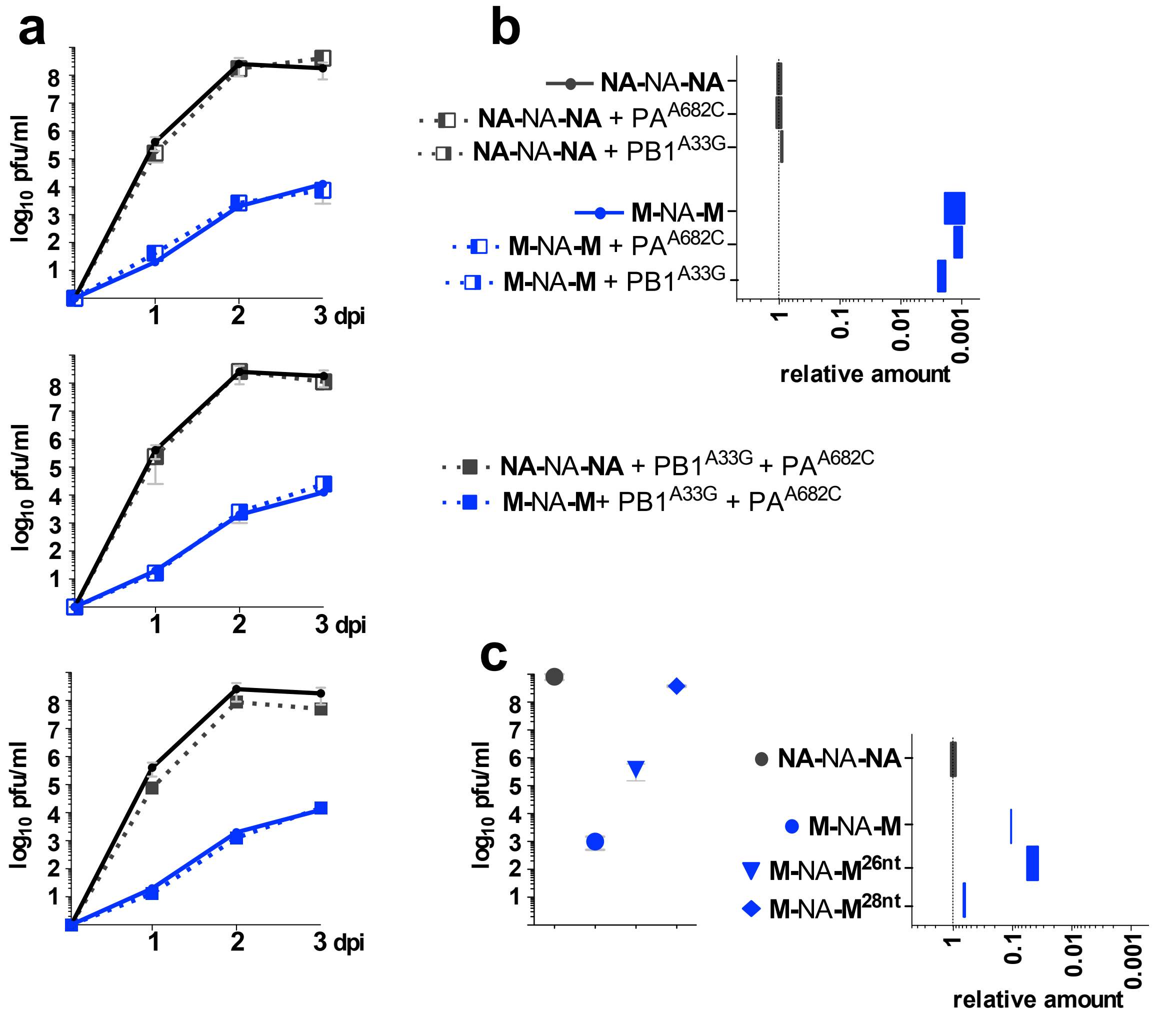

Supplementary Figure S6.

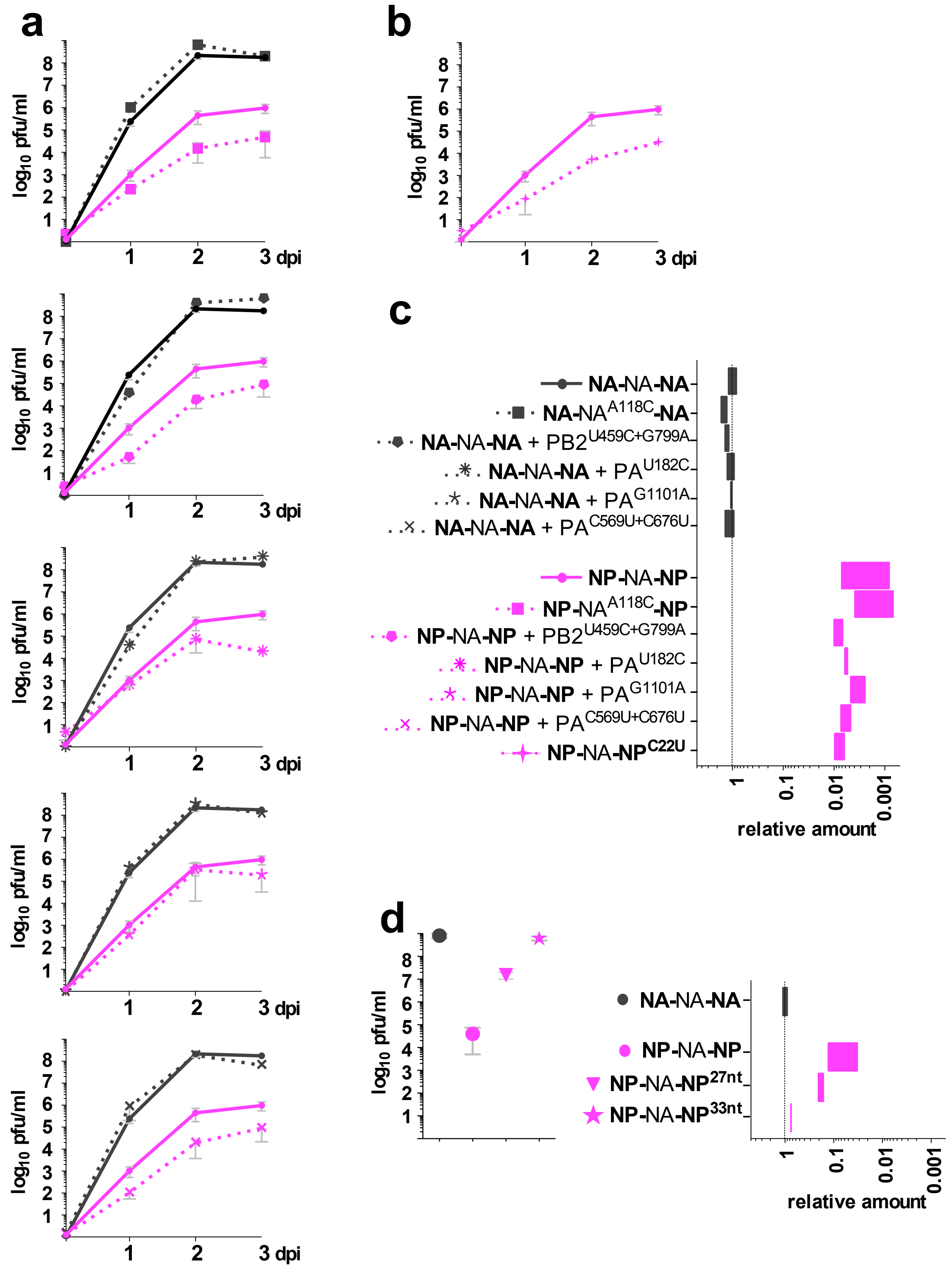

Supplementary Figure S7.

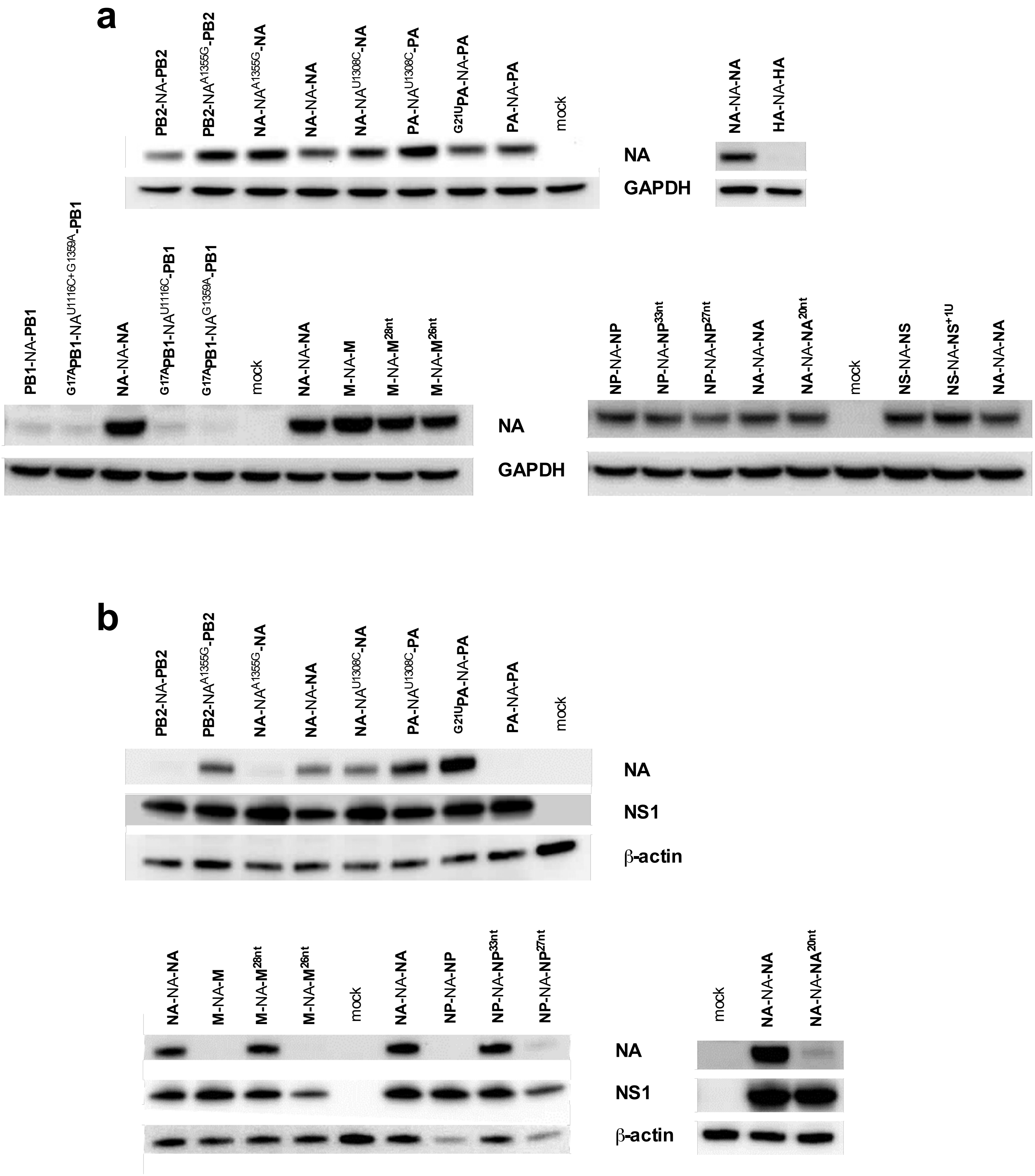

Supplementary Figure S8.

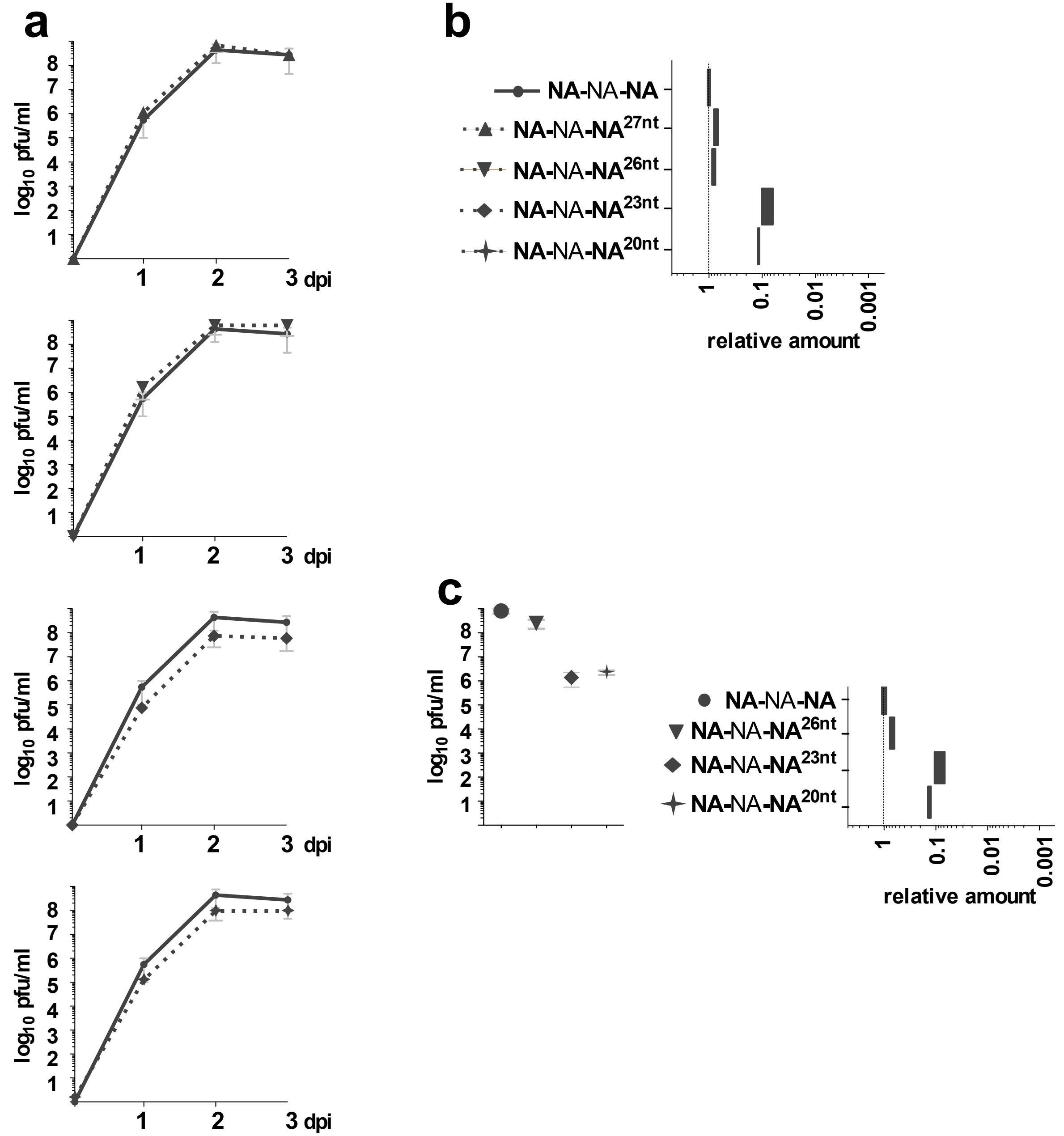

Supplementary Figure S9.

Supplementary Table S1: Genomic vRNA sequences of the 5' and 3' NC regions and mutations introduced in the X-NA-X viruses produced by reverse genetics

**3'X**-NA-**X5'**[a] reverse genetics viruses	vRNA 5'NC length (nt)	vRNA 5'NC sequence[b]	NA coding sequence[c] nt (aa)	vRNA 3'NC sequence[b]	vRNA 3'NC length (nt)	coding sequence[d] segment-nt (aa)
**PB2**-NA-**PB2**	34	AGUAGAAACAAGGUCGUUUUUAAACUAUUCGACA*CUA*		*CAU*AUUGAAUAUAAUUGACCUGCUUUCGCU	27	
**PB2**-NA-**PB2**	34	AGUAGAAACAAGGUCGUUUUUAAACUAUUCGACA*CUA*		*CAU*AUUGAAUAUAAUUGACCUGCUUUCGCU	27	HA-A1145G (K382R)
**PB2**-NA^{A1355G}-**PB2**	34	AGUAGAAACAAGGUCGUUUUUAAACUAUUCGACA*CUA*	NA-A1355G (D452G)	*CAU*AUUGAAUAUAAUUGACCUGCUUUCGCU	27	
**PB2**-NA^{A1355C}-**PB2**	34	AGUAGAAACAAGGUCGUUUUUAAACUAUUCGACA*CUA*	NA-A1355C (D452A)	*CAU*AUUGAAUAUAAUUGACCUGCUUUCGCU	27	
**PB2**-NA^{A1355U}-**PB2**	34	AGUAGAAACAAGGUCGUUUUUAAACUAUUCGACA*CUA*	NA-A1355U (D452V)	*CAU*AUUGAAUAUAAUUGACCUGCUUUCGCU	27	
**NA**-NA^{A1355G}-**NA**	28	AGUAGAAACAAGGAGUUUUUUGAACAAA*CUA*	NA-A1355G (D452G)	*CAU*UAAACUCCUGCUUUUGCU	19	
**NA**-NA-**NA**	28	AGUAGAAACAAGGAGUUUUUUGAACAAA*CUA*		*CAU*UAAACUCCUGCUUUUGCU	19	HA-A1145G (K382R)
**PB1**-NA-**PB1**	43	AGUAGAAACAAGGCAUUUUUUCAUGAAGGACAAGCUAAAUUCA*CUA*		*CAU*CAAAUGGUUUGCCUGCUUUCGCU	24	
**PB1**-NA-**PB1**	43	AGUAGAAACAAGGCAUUUUUUCAUGAAGGACAAGCUAAAUUCA*CUA*		*CAU*CAAAUGGUUUGCCUGCUUUCGCU	24	PB1-U1572C (-)
^{G17A}**PB1**-NA-**PB1**	43	AGUAGAAACAAGGCAUUUUUUCAUGAAGGACAAGCUAAAUUCA*CUA*		*CAU*CAAAUG**A**UUUGCCUGCUUUCGCU	24	
^{G17A}**PB1**-NA^{U1116C}-**PB1**	43	AGUAGAAACAAGGCAUUUUUUCAUGAAGGACAAGCUAAAUUCA*CUA*	NA-U1116C (-)	*CAU*CAAAUG**A**UUUGCCUGCUUUCGCU	24	
^{G17A}**PB1**-NA^{G1359A}-**PB1**	43	AGUAGAAACAAGGCAUUUUUUCAUGAAGGACAAGCUAAAUUCA*CUA*	NA-G1359A (-)	*CAU*CAAAUG**A**UUUGCCUGCUUUCGCU	24	
^{G17A}**PB1**-NA$^{U1116C+G1359A}$-**PB1**	43	AGUAGAAACAAGGCAUUUUUUCAUGAAGGACAAGCUAAAUUCA*CUA*	NA-U1116C (-) + NA-G1359A (-)	*CAU*CAAAUG**A**UUUGCCUGCUUUCGCU	24	
**NA**-NA-**NA**	28	AGUAGAAACAAGGAGUUUUUUGAACAAA*CUA*		*CAU*UAAACUCCUGCUUUUGCU	19	PB1-U1572C (-)
**PA**-NA-**PA**	58	AGUAGAAACAAGGUACUUUUUUGGACAGUAUGGAUAGCAAAUAGUAGCAUUGCCACAA*CUA*		*CAU*UUGAAUCAGUACCUGCUUUCGCU	24	
**PA**-NA-**PA**	58	AGUAGAAACAAGGUACUUUUUUGGACAGUAUGGAUAGCAAAUAGUAGCAUUGCCACAA*CUA*		*CAU*UUGAAUCAGUACCUGCUUUCGCU	24	PA-G1809A (-)
^{G21U}**PA**-NA-**PA**	58	AGUAGAAACAAGGUACUUUUUUGGACAGUAUGGAUAGCAAAUAGUAGCAUUGCCACAA*CUA*		*CAU*UU**U**AAUCAGUACCUGCUUUCGCU	24	
**PA**-NA^{U1308C}-**PA**	58	AGUAGAAACAAGGUACUUUUUUGGACAGUAUGGAUAGCAAAUAGUAGCAUUGCCACAA*CUA*	NA-U1308C (-)	*CAU*UUGAAUCAGUACCUGCUUUCGCU	24	
^{G21U}**PA**-NA^{U1308C}-**PA**	58	AGUAGAAACAAGGUACUUUUUUGGACAGUAUGGAUAGCAAAUAGUAGCAUUGCCACAA*CUA*	NA-U1308C (-)	*CAU*UU**U**AAUCAGUACCUGCUUUCGCU	24	
**NA**-NA^{U1308C}-**NA**	28	AGUAGAAACAAGGAGUUUUUUGAACAAA*CUA*	NA-U1308C (-)	*CAU*UAAACUCCUGCUUUUGCU	19	
**NA**-NA-**NA**	28	AGUAGAAACAAGGAGUUUUUUGAACAAA*CUA*		*CAU*UAAACUCCUGCUUUUGCU	19	PA-G1809A (-)
**HA**-NA-**HA**	45	AGUAGAAACAAGGGUGUUUUUCCUUAUAUUUCUGAAAUCCUAAUC*CUA*		*CAU*UUGGUUGUUUUUAUUUUCCCCUGCUUUUGCU	32	
**NP**-NA-**NP**	23	AGUAGAAACAAGGGUAUUUUUCU*CUA*		*CAU*GAUUUCGAUGUCACUCUGUGAGUGAUUAUCUACCCUGCUUUUGCU	45	
**NP**-NA-**NP**	23	AGUAGAAACAAGGGUAUUUUUCU*CUA*		*CAU*GAUUUCGAUGUCACUCUGUGAGUGAUUAUCUACCCUGCUUUUGCU	45	PB2-U459C (-) + G799A (V267I)
**NP**-NA-**NP**	23	AGUAGAAACAAGGGUAUUUUUCU*CUA*		*CAU*GAUUUCGAUGUCACUCUGUGAGUGAUUAUCUACCCUGCUUUUGCU	45	PA-U182C (I61T)
**NP**-NA-**NP**	23	AGUAGAAACAAGGGUAUUUUUCU*CUA*		*CAU*GAUUUCGAUGUCACUCUGUGAGUGAUUAUCUACCCUGCUUUUGCU	45	PA-G1101A (-)
**NP**-NA-**NP**	23	AGUAGAAACAAGGGUAUUUUUCU*CUA*		*CAU*GAUUUCGAUGUCACUCUGUGAGUGAUUAUCUACCCUGCUUUUGCU	45	PA-C569U (S190F) + C676U (L226F)
**NP**-NA-**NP**^{C22U}	23	AGUAGAAACAAGGGUAUUUUU**U**U*CUA*		*CAU*GAUUUCGAUGUCACUCUGUGAGUGAUUAUCUACCCUGCUUUUGCU	45	
**NP**-NA^{A118C}-**NP**	23	AGUAGAAACAAGGGUAUUUUUCU*CUA*	NA-A118C (T40P)	*CAU*GAUUUCGAUGUCACUCUGUGAGUGAUUAUCUACCCUGCUUUUGCU	45	
**NP**-NA-**NP**^{24nt}	24	AGUAGAAACAAGGGUAUUUUU**U**U**C***CUA*		*CAU*GAUUUCGAUGUCACUCUGUGAGUGAUUAUCUACCCUGCUUUUGCU	45	
**NP**-NA-**NP**^{27nt}	27	AGUAGAAACAAGGGUAUUUUU**U**U**CUCU***CUA*		*CAU*GAUUUCGAUGUCACUCUGUGAGUGAUUAUCUACCCUGCUUUUGCU	45	
**NP**-NA-**NP**^{28nt}	28	AGUAGAAACAAGGGUAUUUUU**U**U**CUAUU***CUA*		*CAU*GAUUUCGAUGUCACUCUGUGAGUGAUUAUCUACCCUGCUUUUGCU	45	
**NP**-NA-**NP**^{33nt}	33	AGUAGAAACAAGGGUAUUUUU**U**U**CUAUUUUUUU***CUA*		*CAU*GAUUUCGAUGUCACUCUGUGAGUGAUUAUCUACCCUGCUUUUGCU	45	
**NA**-NA^{A118C}-**NA**	28	AGUAGAAACAAGGAGUUUUUUGAACAAA*CUA*	NA-A118C (T40P)	*CAU*UAAACUCCUGCUUUUGCU	19	
**NA**-NA-**NA**	28	AGUAGAAACAAGGAGUUUUUUGAACAAA*CUA*		*CAU*UAAACUCCUGCUUUUGCU	19	PB2-U459C (-) + G799A (V267I)
**NA**-NA-**NA**	28	AGUAGAAACAAGGAGUUUUUUGAACAAA*CUA*		*CAU*UAAACUCCUGCUUUUGCU	19	PA-U182C (I61T)
**NA**-NA-**NA**	28	AGUAGAAACAAGGAGUUUUUUGAACAAA*CUA*		*CAU*UAAACUCCUGCUUUUGCU	19	PA-G1101A (-)
**NA**-NA-**NA**	28	AGUAGAAACAAGGAGUUUUUUGAACAAA*CUA*		*CAU*UAAACUCCUGCUUUUGCU	19	PA-C569U (S190F) + C676U (L226F)
**NA**-NA-**NA**	28	AGUAGAAACAAGGAGUUUUUUGAACAAA*CUA*		*CAU*UAAACUCCUGCUUUUGCU	19	
**NA**-NA-**NA**^{27nt}	27	AGUAGAAACAAGGAGUUUUUUGAACAA*CUA*		*CAU*UAAACUCCUGCUUUUGCU	19	
**NA**-NA-**NA**^{26nt}	26	AGUAGAAACAAGGAGUUUUUUGAACA*CUA*		*CAU*UAAACUCCUGCUUUUGCU	19	
**NA**-NA-**NA**^{23nt}	23	AGUAGAAACAAGGAGUUUUUUGA*CUA*		*CAU*UAAACUCCUGCUUUUGCU	19	
**NA**-NA-**NA**^{20nt}	20	AGUAGAAACAAGGAGUUUUU*UCA*		*CAU*UAAACUCCUGCUUUUGCU	19	
**M**-NA-**Mi**	20	AGUAGAAACAAGGUAGUUUU*CUA*		*CAU*CUUUCAAUAUCUACCUGCUUUCGCU	25	
**M**-NA-**M**	20	AGUAGAAACAAGGUAGUUUU***U****UA*		*CAU*CUUUCAAUAUCUACCUGCUUUCGCU	25	
**M**-NA-**M**	20	AGUAGAAACAAGGUAGUUUU***U****UA*		*CAU*CUUUCAAUAUCUACCUGCUUUCGCU	25	PB1-A33G (-)
**M**-NA-**M**	20	AGUAGAAACAAGGUAGUUUU***U****UA*		*CAU*CUUUCAAUAUCUACCUGCUUUCGCU	25	PA-A682C (N228H)
**M**-NA-**M**	20	AGUAGAAACAAGGUAGUUUU***U****UA*		*CAU*CUUUCAAUAUCUACCUGCUUUCGCU	25	PB1-A33G (-) +PA-A682C (N228H)
**M**-NA-**M**^{24nt}	24	AGUAGAAACAAGGUAGUUUU**U**UA**U***UUA*		*CAU*CUUUCAAUAUCUACCUGCUUUCGCU	25	
**M**-NA-**M**^{26nt}	26	AGUAGAAACAAGGUAGUUUU**U**UA**UUU***UUA*		*CAU*CUUUCAAUAUCUACCUGCUUUCGCU	25	
**M**-NA-**M**^{28nt}	28	AGUAGAAACAAGGUAGUUUU**U**UA**GUUUU***UUA*		*CAU*CUUUCAAUAUCUACCUGCUUUCGCU	25	
**NA**-NA-**NA**	28	AGUAGAAACAAGGAGUUUUUUGAACAAA*CUA*		*CAU*UAAACUCCUGCUUUUGCU	19	PB1-A33G (-)
**NA**-NA-**NA**	28	AGUAGAAACAAGGAGUUUUUUGAACAAA*CUA*		*CAU*UAAACUCCUGCUUUUGCU	19	PA-A682C (N228H)
**NA**-NA-**NA**	28	AGUAGAAACAAGGAGUUUUUUGAACAAA*CUA*		*CAU*UAAACUCCUGCUUUUGCU	19	PB1-A33G (-) +PA-A682C (N228H)
**NS**-NA-**NS**	26	AGUAGAAACAAGGGUGUUUUUUAUUA*CUA*		*CAU*UAUGUCUUUGUCACCCUGCUUUUGCU	26	
**NS**-NA-**NS**$^{+1U}$	27	AGUAGAAACAAGGGUGUUUUUU**U**AUUA*CUA*		*CAU*UAUGUCUUUGUCACCCUGCUUUUGCU	26	
**NS**-NA^{G993A}-**NS**	26	AGUAGAAACAAGGGUGUUUUUUAUUA*CUA*	NA-G993A (-)	*CAU*UAUGUCUUUGUCACCCUGCUUUUGCU	26	
**NA**-NA^{G993A}-**NA**	28	AGUAGAAACAAGGAGUUUUUUGAACAAA*CUA*	NA-G993A (-)	*CAU*UAAACUCCUGCUUUUGCU	19	

a: name of virus produced by reverse genetics
b: nucleotide substitutions or insertions are in bold; start and stop codons are in italics
c: mutations in the NA coding region, in brackets amino acid substitution; (-) indicates a synonymous mutation
d: mutations in the coding region of the indicated segments; in brackets amino acid substitution; (-) indicates a synonymous mutation
